# The (Anti)aromatic Properties of Cyclo[n]Carbons: Myth or Reality?

**DOI:** 10.1002/jcc.70283

**Published:** 2025-11-29

**Authors:** O. A. Stasyuk, G. George, C. Curutchet, F. Plasser, A. J. Stasyuk

**Affiliations:** ^1^ Institut de Química Computacional i Catàlisi and Departament de Química Universitat de Girona Girona Spain; ^2^ Departament de Farmàcia i Tecnologia Farmacèutica, i Fisicoquímica, Facultat de Farmàcia i Ciències de l'Alimentació Universitat de Barcelona (UB) Barcelona Spain; ^3^ Institut de Química Teòrica i Computacional (IQTCUB), Universitat de Barcelona (UB) Barcelona Spain; ^4^ Department of Chemistry Loughborough University Loughborough UK; ^5^ Faculty of Chemistry University of Warsaw Warsaw Poland

**Keywords:** aromatic stabilization energy, aromaticity, cyclo[n]carbons, EDDB, electron affinity, electron delocalization

## Abstract

Recent advances in on‐surface chemistry have enabled the synthesis and structural characterization of even‐numbered cyclo[n]carbons, traditionally classified as either doubly aromatic (*n* = 4k + 2) or doubly antiaromatic (*n* = 4k) based on their in‐plane and out‐of‐plane π‐electron circuits. However, recent studies have increasingly questioned this classification, suggesting instead that these molecules are more accurately described as non‐aromatic. In this work, we computationally examine the electron affinities and (anti)aromatic character of cyclo[n]carbons with *n* = 16–30 using energetic, structural, and electronic aromaticity descriptors. Adiabatic electron affinity (AEA) analysis reveals a high degree of uniformity across the series of both nominally aromatic and antiaromatic members. Aromatic stabilization energy (ASE) values, derived from homodesmotic and disproportionation reactions, indicate slight destabilization only for C_16_ and C_20_, and low stabilization for the remaining systems. In particular, ASE is less than 2 kcal/mol for cyclo[n]carbons with *n* ≥ 24. This suggests that neither aromatic nor antiaromatic character significantly contributes to the thermodynamic stability of larger cyclocarbons. EDDB analysis further supports this conclusion, with only about 22%–27% of π‐electrons participating in delocalization. While delocalization is slightly greater in cyclo[n]carbons with *n* = 4k + 2, the difference diminishes with increasing size. Upon two‐electron reduction to the dianionic state, all cyclo[n]carbons exhibit bond length equalization and increased delocalization. These results suggest that only small cyclo[n]carbons (*n* < 24) can be classified as weakly (anti)aromatic, while larger cyclo[n]carbons (*n* ≥ 24) are more appropriately classified as non‐aromatic systems. The aromaticity of all considered cyclocarbons becomes more pronounced in corresponding dianionic forms due to cooperative structural and electronic effects. Thus, this work provides a unified framework for interpreting and predicting the electronic behavior of cyclocarbons.

## Graphical abstract

Small neutral cyclo[n]carbons (*n* < 24) possess only weak aromatic or antiaromatic character, with low stabilization energies and moderate electron delocalization, while the characteristics of larger systems allow them to be classified as non‐aromatic.

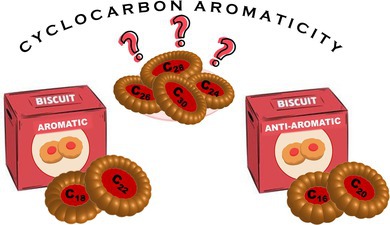

## 1 Introduction

Cyclo[n]carbons represent one of the most recently discovered carbon allotropes, which are molecular rings made up of n carbon atoms. The existence of this unique carbon structure was initially proposed by Pitzer and Clementi in 1959 [1]. Guided by Hückel's rule, they suggested that cyclo[n]carbons with *n* = 4k + 2 atoms, where k is a non‐negative integer, would be stable. Only 30 years later, in 1989, Diederich et al. provided the first experimental evidence of the existence of cyclo[18]carbon [2]. Despite this breakthrough, the high reactivity of cyclo[n]carbons prevented scientists from isolating and further studying their properties. Motivated by several scientific reports confirming the presence of cyclo[18]carbon in the gas phase [3, 4, 5], researchers have performed numerous quantum‐mechanical calculations to predict its structure. As it turned out, the cyclo[18]carbon or C_18_ molecule, composed of sp‐hybridized carbon atoms, could exist in either polyynic or cumulenic forms. Importantly, the relative stability of one or another structure depends on the computational method used for prediction [6, 7, 8, 9, 10, 11, 12]. It took another 30 years to experimentally verify its structure. In 2019, Kaiser et al. successfully synthesized and characterized cyclo[18]carbon using atomic force microscopy (AFM) and scanning tunneling microscopy (STM) [13]. Their findings confirmed that C_18_ has a polyynic structure with alternating bond lengths. Since then, the ultra‐inert conditions and low temperatures used to synthesize cyclo[n]carbons via tip‐induced on‐surface chemistry have enabled the synthesis of seven even‐numbered cyclo[n]carbons, where *n* = 10, 12, 14, 16, 18, 20, and 26, as well as one odd‐numbered cyclo[13]carbon [13, 14, 15, 16].

It is generally assumed that even‐numbered cyclo[n]carbons can be doubly aromatic (for *n* = (4k + 2) = 10, 14, 18, etc.) or doubly antiaromatic (for *n* = 4k = 12, 16, 20, etc.) due to the presence of two perpendicular π‐electron systems (one out‐of‐plane and one in‐plane). Various descriptors have been used to characterize the aromatic/antiaromatic nature of cyclo[n]carbons [17, 18, 19, 20, 21]. In particular, nucleus‐independent chemical shifts (NICS) [22, 23], isotropic chemical shielding surface (ICSS) [24], gauge‐including magnetically induced current (GIMIC) [25, 26], and anisotropy of current‐induced density (ACID) [27, 28] methods have consistently confirmed the strong aromatic/antiaromatic characteristics of the corresponding cyclo[n]carbons. Baryshnikov et al., based on the GIMIC results, demonstrated that the Hückel rules of aromaticity and antiaromaticity hold true for the rings smaller than C_32_—C_34_, while larger rings, such as C_50_ and above, are nonaromatic, with no evidence of global delocalization [17]. GIMIC and ACID analyses demonstrated that the ring currents in these molecules originate from both in‐plane and out‐of‐plane π‐electrons, supporting the concept of double aromaticity in these systems [11, 13, 17, 29].

Lately, a growing number of studies have suggested that cyclocarbons should be classified as non‐aromatic molecules. In a series of recent publications, Baranac‐Stojanović analyzed various properties of even‐numbered cyclo[n]carbons with *n* = 6–24 [30, 31]. Several structural parameters, including bond order, bond length and bond angle alternation, magnetic shielding, and electronic descriptors such as the aromatic fluctuation index (FLU) and electron density of delocalized bonds (EDDB) were considered. The results show that, starting from cyclo[16]carbon, both in‐plane and out‐of‐plane π‐electron systems of cyclocarbons behave in a similar way. Their energetic stabilization is comparable to that of acyclic polyynes. This indicates that cyclocarbons are almost unaffected by (anti)aromaticity effects. Such conclusion is further supported by a study by Solà and Szczepanik [32], who, based on the particle on a ring model, noted that while electron delocalization may reduce kinetic energy and stabilize small cyclic systems, in larger rings such as cyclo[18]carbon this effect is offset by an increase in the exchange correlation energy between electrons of the same spin. This leads to a preference for localized bonds and supports the classification of such molecules as non‐aromatic.

In our recent theoretical studies, we explored the van der Waals (vdW) complexes formed between cyclo[16]carbon (C_16_) and cyclo[18]carbon (C_18_) with representative electron‐donor and electron‐acceptor species [33]. As expected, comparison of their frontier molecular orbitals revealed that the HOMO‐LUMO (HL) gap for the antiaromatic C_16_ molecule is smaller than that of the aromatic C_18_ molecule. Interestingly, however, the computed adiabatic electron affinities (AEAs) of both cyclocarbons were found to be quite similar. Such similarity in AEAs seems unusual, given that antiaromatic molecules typically exhibit higher electron affinities due to the inherent destabilization of their neutral form, which is partially relieved upon reduction to the radical anion. Motivated by this observation, along with recent insights into the (anti)aromatic character of various cyclocarbons, we conducted a more detailed investigation of aromaticity trends across the cyclo[n]carbon series. For this purpose, we selected four molecules previously classified as aromatic (C_18_, C_22_, C_26_, and C_30_) and four as antiaromatic (C_16_, C_20_, C_24_, and C_28_), and assessed their (anti)aromatic nature using a range of energetic, structural, and electronic aromaticity descriptors.

## 2 Results and Discussion

### 2.1 Adiabatic Electron Affinities

Previously, we demonstrated that the range‐separated ωB97X‐D functional [34] with damped atom‐atom dispersion corrections accurately predicts the geometric structure and electronic properties of cyclo[n]carbons [33]. Given that the AEA calculations include anionic species, the chosen ωB97X‐D functional was used in combination with the ma‐def2‐TZVPP basis set [35], which contains polarization and diffuse functions. The structures of neutral, monoanionic, and dianionic species were optimized without any constraints using Gaussian 16 (rev. C01) [36]. Normal mode vibrational frequencies were also calculated for each system to confirm the presence of a local minimum on the potential energy surface at the same level of theory. For detailed methodology, see Supporting Information.

Considering that all reported cyclo[n]carbons were obtained in an isolated state (on the bilayer NaCl/Cu(111) surface), we first calculated their first (AEA1) and second (AEA2) adiabatic electron affinities in the gas phase (Table S1). As shown in Table 1, the predicted AEA1 values increase monotonically with the size of cyclo[n]carbons meaning that, in vacuum, they become stronger electron acceptors as the ring gets larger. For the smallest molecule considered C_16_, AEA1 amounts to 2.49 eV, while for the largest C_30_, it increases to 3.01 eV. The behavior of the AEA2 follows the same trend, showing a monotonic increase with the molecule size. Unexpectedly, the AEA2 values for C_16_, C_18_, C_20_ and C_22_ cyclocarbons were found to be negative (Table 1). Following previous experience on carbon macrocycles [37], we note that the formation of multiply charged anions in vacuum is expected to be unfavorable, and that dielectric screening by the environment is needed to stabilize the additional charges. Providing an accurate representation of the NaCl/Cu(111) surface is challenging; instead, we employ a conductor‐like polarizable continuum model (CPCM) [38] using a dielectric constant of *ε* = 3.0 to represent an averaged screening effect. Such a model is certainly not an accurate representation of an NaCl/Cu(111) surface, but it fulfills its main role of consistently providing stabilization to the dianionic states. Indeed, the approach resulted in a significant shift in AEA values, yielding positive AEA2 for all systems. Interestingly, using this approach the AEA values also become more uniform and the enhancement with larger cyclocarbon rings becomes weaker.

**TABLE 1:** First and second adiabatic electron affinities (AEA1 and AEA2) calculated for a series of cyclo[n]carbons in gas phase (VAC) and within CPCM solvation model using a generic low‐polarity solvent (*ε* = 3.0).^[^ ^a^ ^,^ ^b^ ^]^

Cyclo[n]carbon	AEA1, eV		AEA2, eV	
	VAC	CPCM	VAC	CPCM
C_16_	2.485	3.349	−1.049 (T)	2.010 (T)
C_18_	2.587	3.341	−0.584 (S)	2.393 (S)
C_20_	2.703	3.377	−0.416 (T)	2.293 (T)
C_22_	2.825	3.373	−0.260 (S)	2.465 (S)
C_24_	2.913	3.389	0.039 (T)	2.517 (T)
C_26_	2.939	3.401	0.216 (T)	2.512 (T)
C_28_	3.112	3.440	0.433 (T)	2.638 (T)
C_30_	3.013	3.423	0.587 (T)	2.675 (T)

^a^ Dianion can be either a closed‐shell singlet or a triplet. The letter T or S in parentheses refers to the lowest energy state.

^b^ $\mathrm{AEA}1={∆G}_{C_{n}^{0}}^{\mathrm{eq}}-{∆G}_{C_{n}^{1-}}^{\mathrm{eq}};\mathrm{AEA}2={∆G}_{C_{n}^{1-}}^{\mathrm{eq}}-{∆G}_{C_{n}^{2-}}^{\mathrm{eq}}$.

**FIGURE 1:**
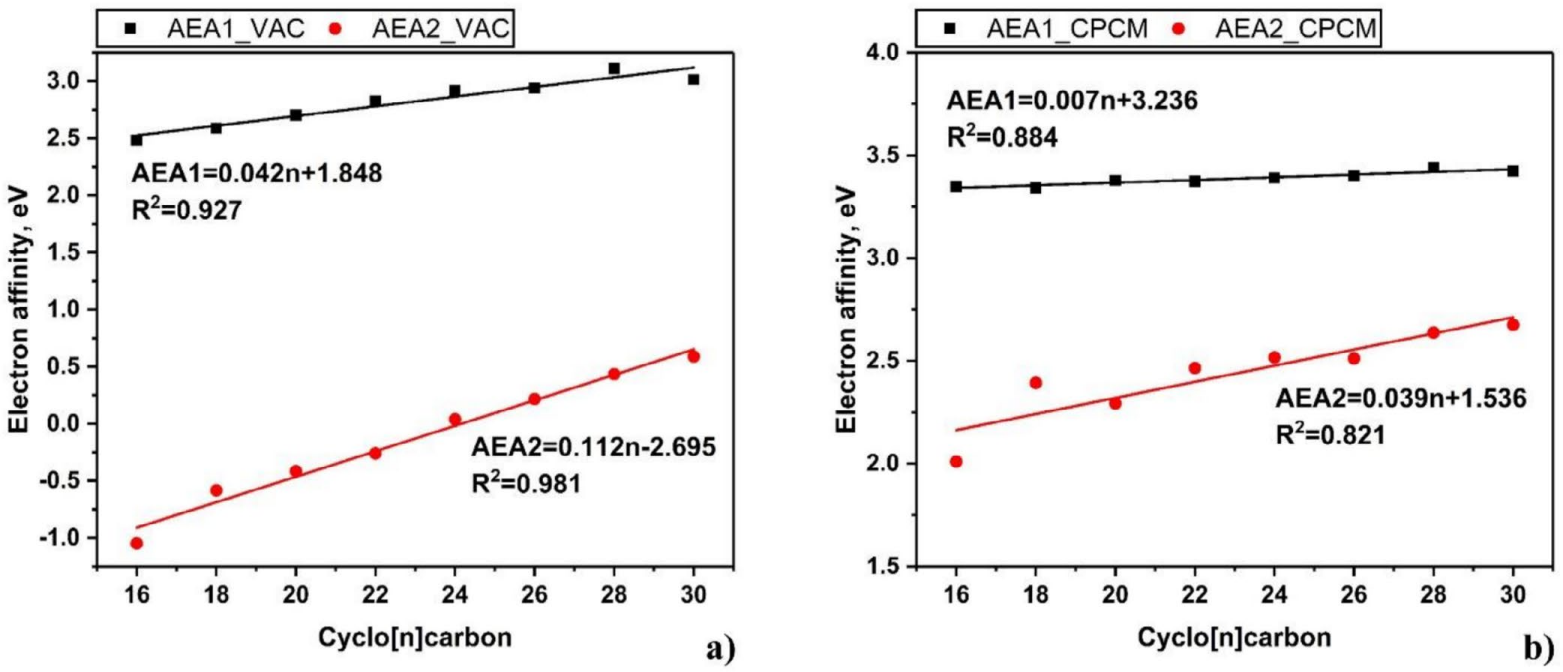
Adiabatic electron affinities for a series of cyclo[n]carbons in the gas phase (a) and within CPCM solvation model (b).

A detailed analysis of the obtained AEA data revealed a high degree of homogeneity. In particular, regression analysis performed for both formally aromatic and antiaromatic cyclo[n]carbons resulted in very similar slope values in linear regression (Figure S1). A two‐sample *t*‐test with a predetermined threshold of 0.001 did not reveal a significant difference between AEA data for aromatic and antiaromatic cyclocarbons (Table S2). Additionally, when data for both types of cyclo[n]carbons were analyzed together, a high correlation coefficient was observed, with *R* ^2^ values ranging from 0.82 to 0.98 (Figure 1). Only the AEA2 values computed using CPCM for the first three systems (*n* = 16, 18, 20) exhibit some alternation anticipated when (anti)aromaticity effects are present.

Usually for systems with notable (anti)aromaticity, the regressions between AEA values and the number of carbon atoms exhibit relatively low *R* ^2^ values, because the stabilities of aromatic and antiaromatic ions also depend on the resonance energy [39]. It is well known that antiaromatic systems such as pentalene, cyclooctatetraene in planar geometry, and cyclobutadiene have abnormally high electron affinities, whereas aromatic systems like benzene, triphenylene, and coronene have unusually low AEAs. This behavior is explained not only by the size of the molecules but also by the stabilization or destabilization of the neutral molecule, which is changed in the radical anion. The lack of substantial differences in AEA behavior for aromatic and antiaromatic cyclo[n]carbons prompted us to examine the phenomenon of aromaticity in cyclo[n]carbons in more detail.

### 2.2 Aromatic Stabilization Energy

Aromaticity is one of the most fundamental concepts in chemistry. However, the absence of a quantum‐mechanical operator that directly extracts aromaticity from the wavefunction introduces ambiguity, resulting in various descriptors, such as geometric, magnetic or electronic, that are used to determine whether a molecule is aromatic or antiaromatic. Although these descriptors have repeatedly demonstrated their applicability in identifying and characterizing aromatic and antiaromatic systems, we believe that the key criterion remains the enhanced energetic stability of aromatic compounds, or the reduced stability of antiaromatic compounds, compared to non‐aromatic ones.

To estimate the energy associated with the stabilization/destabilization of aromatic/antiaromatic cyclo[n]carbons, we calculated the aromatic stabilization energy (ASE) [40, 41, 42], which should be positive for aromatic molecules and negative for antiaromatic ones. ASE can be estimated by constructing a thermodynamic cycle for a hypothetical isodesmic reaction that disrupts the π‐conjugation in the studied cyclic system. However, uncertainty in ASE values arises because different thermodynamic cycles may yield different results [43, 44, 45]. Ideally, reactions should ensure an equal number of C—C bond types and an equal number of each type of carbon atom in both the reactants and products [46].

**FIGURE 2:**
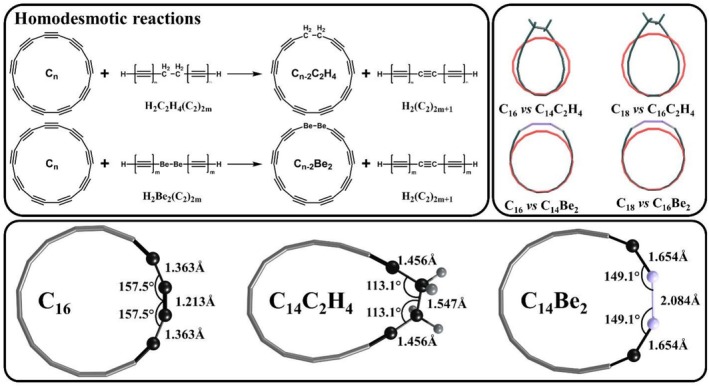
Homodesmotic reaction considered for evaluation of cyclo[n]carbons ASE, along with selected geometrical parameters for C_16_ cyclocarbon and its derivatives.

**FIGURE 3:**
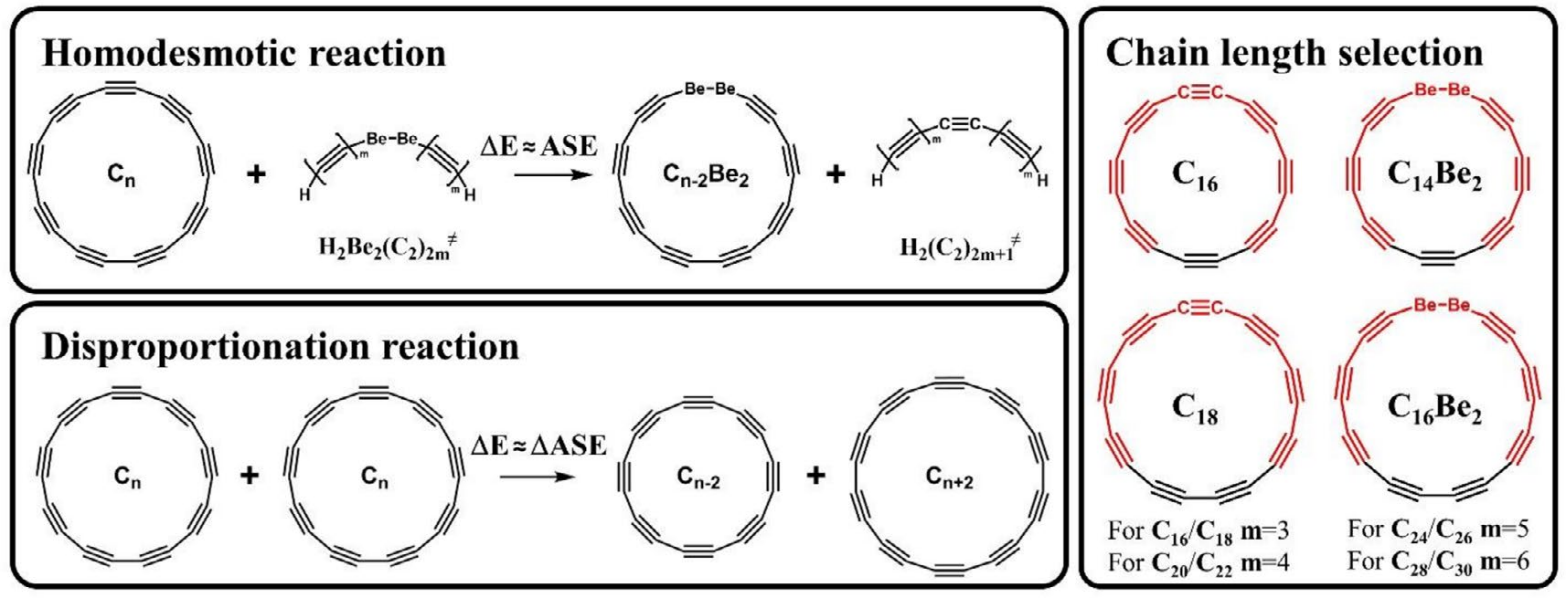
Modified homodesmotic and disproportionation reactions used to evaluate and validate the ASE of cyclo[n]carbons, along with the selection of appropriate polyyne chain length, using C_16_ and C_18_ cyclocarbons as examples. The molecular geometries of cyclocarbons were optimized without constraints using the ωB97X‐D/ma‐def2‐TZVPP level of theory, and single‐point energies for the curved polyynes were calculated at the same level.

Initially, we assumed that replacing an acetylenic (—C≡C—) fragment with an ethane (—CH_2_—CH_2_—) unit would be sufficient for determining ASE. However, this replacement caused dramatic geometric distortions, including in‐plane elongation and out‐of‐plane tilting, compared to pristine cyclocarbons (Figure 2). After an extensive literature search, we found another candidate that could potentially replace the acetylenic unit without causing significant geometric distortion. In particular, several years ago, Schaefer and co‐workers demonstrated that linear structures for beryllium (Be) and magnesium (Mg) dihydrides are the global minimum structures [47]. Thus, we decided to try replacing one acetylenic fragment in each cyclocarbon with a —Be—Be— bond. In addition, we studied a formal disproportionation‐type reaction, in which each cyclo[n]carbon transforms into smaller and larger members of the cyclocarbon family (Figure 3).

Although the geometrical distortions caused by replacing the acetylenic unit with a —Be—Be— fragment were much smaller than those caused by the ethane fragment, the structural differences between pristine cyclocarbons and their Be‐derivatives are still too significant to assume comparable strain energies for C_n_ and C_n−2_Be_2_ molecules (Figures S2 and S3). It is important to note that, for accurate estimation of ASE using the homodesmotic reaction approach, the equality of strain energies should be maintained not for each individual species in the reaction, but for the total strain energy of all the products and reactants combined. With this in mind, we modified the proposed homodesmotic reaction, considering H_2_Be_2_(C_2_)_2m_ and H_2_(C_2_)_2m+1_ structures not as linear chains but as curved structures, with curvature precisely matching that of the corresponding fragment in the cyclo[n]carbons and their Be‐derivatives (Figure 3).

In all cases, the length of the curved polyyne chains was chosen to maximize structural similarity to the cyclocarbons. In particular, for C_16_, C_18_ and their corresponding Be‐derivatives, the curved polyyne structures H_2_Be_2_(C_2_)_2m_ and H_2_(C_2_)_2m+1_ with *m* = 3 are more suitable. It is important to note that while *m* = 4 could be considered for the C_18_ cyclocarbon, this would lead to spatial overlap of the terminal hydrogen atoms. The m values used for each cyclocarbon of interest are indicated in Figure 3.

Since disruption of π‐electron delocalization in a system is a prerequisite for the reaction used to calculate ASE, we visually compared the HOMOs for both aromatic and antiaromatic C_n_ cyclocarbons and their corresponding C_n−2_Be_2_ derivatives. The results clearly demonstrate the disruption of both in‐plane and out‐of‐plane π‐conjugated systems in all cases. In the Be‐substituted cyclocarbons, the HOMO orbitals are not uniformly distributed across the entire ring, but a gap appears in the region of the —Be—Be— fragment (Figure S4).

The ASE values calculated for all cyclo[n]carbons are summarized in Table 2. The values derived from the proposed homodesmotic reaction are slightly negative for only two systems of interest: the formally antiaromatic C_16_ and C_20_ cyclocarbons, with values of −3.43 and −0.59 kcal/mol, respectively, indicating thermodynamic destabilization relative to the corresponding nonaromatic reference structure. All other ASE values are positive (indicative of aromaticity), ranging from 0.30 to 6.57 kcal/mol.

**TABLE 2:** Aromatic stabilization energies (ASE, in kcal/mol) and their difference (ΔASE, in kcal/mol) calculated from the enthalpies of formation for a series of cyclo[n]carbons using homodesmotic and disproportionation reactions.

	Cyclo[n]carbons							
	C_16_	C_18_	C_20_	C_22_	C_24_	C_26_	C_28_	C_30_
Homodesmotic reaction (HHDR)								
ASE^HHDR [^ ^a^ ^]^	−3.43	6.57	−0.59	3.39	0.30	1.98	0.54	1.27
ASE^Norm [^ ^b^ ^]^	−0.107	0.183	−0.015	0.077	0.006	0.038	0.010	0.021
Disproportionation reaction (DR)								
ΔASE^DR [^ ^c^ ^]^	−22.03	15.58	−8.05	6.60	−3.10	2.92	−1.14	1.33
ΔASE^HHDR [^ ^d^ ^]^	−24.79	17.16	−11.15	7.08	−4.77	3.12	−2.16	0.55

^a^ ${\mathrm{ASE}}_{C_{n}}^{\text{HHDR}}=\Delta H_{r}^{\text{HHDR}}=\left(\Delta H_{C_{n-2}{\mathrm{Be}}_{2}}+\Delta H_{H_{2}{\left(C_{2}\right)}_{9}}\right)-\left(\Delta H_{C_{n}}+\Delta H_{H_{2}{\mathrm{Be}}_{2}{\left(C_{2}\right)}_{8}}\right)$.

^b^ ${\mathrm{ASE}}^{\text{Norm}}={\mathrm{ASE}}^{\text{HHDR}}/\mathrm{n\pi}$.

^c^ $\Delta {\mathrm{ASE}}^{\mathrm{DR}}=\Delta H_{r}^{\mathrm{DR}}=\left(\Delta H_{C_{n}}+\Delta H_{C_{n}}\right)-\left(\Delta H_{C_{n-2}}+\Delta H_{C_{n+2}}\right)$.

^d^ $\Delta {\mathrm{ASE}}^{\text{HHDR}}=\left({\mathrm{ASE}}_{C_{n}}^{\text{HHDR}}+{\mathrm{ASE}}_{C_{n}}^{\text{HHDR}}\right)-\left({\mathrm{ASE}}_{C_{n-2}}^{\text{HHDR}}+{\mathrm{ASE}}_{C_{n+2}}^{\text{HHDR}}\right)$.

The positive ASE values obtained for C_18_, C_22_, C_26_, and C_30_ cyclocarbons support their aromatic nature. Conversely, the only very slightly negative and even positive ASE values obtained for C_16_, C_20_, C_24_, and C_28_ molecules do not support their assignment as antiaromatic. In general, the ASE values for all systems are relatively small, indicating the low (anti)aromaticity of the studied cyclocarbons. For comparison with typical benzenoid hydrocarbons, Ciesielski, Cyranski, and co‐workers found that the ASE values vary from 29 to 46 kcal/mol for the systems ranging from benzene to coronene [44, 48]. Importantly, they demonstrated that the ASE per π‐electron (ASE^Norm^) never dropped below 2 kcal/mol [48]. In contrast, for the most aromatic C_18_ cyclocarbon, this ratio is only 0.18 kcal/mol.

To ensure that the results were not influenced by significant changes in hybridization, strain energy, or other factors due to the homodesmotic reaction employed, we also considered the disproportionation reaction (Figure 3). Since aromatic and antiaromatic cyclo[n]carbons alternate, this reaction converts two cyclo[n]carbons of the same nature (aromatic or antiaromatic) into cyclo[n−2]carbon and cyclo[*n* + 2]carbon, which are of opposite nature. Although the disproportionation reaction cannot predict the ASE for a specific cyclo[n]carbon, as it does not break the cyclic π‐conjugation, it can effectively characterize the differences in aromatic stabilization energies (ΔASE^DR^) between the cyclo[n]carbons involved, while ensuring no changes in hybridization, strain energy, or other important factors. A comparison of ΔASE^DR^ values from the disproportionation reaction with those from the homodesmotic reaction (ΔASE^HHDR^) provided in Table 2 revealed the great similarity of the values. This consistency demonstrates the reliability of the ASE results obtained from the homodesmotic reaction. In this context it is worth noting that the ΔASE values consistently show a negative/positive alternation for the 4n/4n + 2 systems, which would, taken by itself, be consistent with antiaromaticity/aromaticity alternation for these systems.

To summarize, we note that C_16_ and C_18_ exhibited thermodynamic destabilization and stabilization, respectively, compared to their non‐aromatic counterparts. However, these properties were weakly pronounced and this effect decreased rapidly as the size of the cyclo[n]carbons increased. From C_24_ onwards, the calculated ASE does not exceed 2 kcal/mol, which suggests classifying them rather as non‐aromatic.

### 2.3 Electronic Criteria of Aromaticity

First, we quantified the cyclic delocalization of π‐electrons in the carbon rings using the electron density of delocalized bonds (EDDB) index [49, 50]. This index reveals the extent of electron delocalization and the portion of electron density that cannot be attributed solely to a particular chemical bond. In typical Hückel 4n + 2 aromatic compounds, the EDDB shows that cyclic delocalization predominates, while in Hückel 4n antiaromatic systems, cyclic delocalization almost completely vanishes. The EDDB demonstrates strong agreement with other widely used aromaticity descriptors, captures similar trends across different aromatic systems and allows visualization of resonance‐stabilized regions [51, 52]. Previously, the EDDB method has been successfully used to analyze aromaticity in large conjugated aromatic and antiaromatic heterocycles [53].

**FIGURE 4:**
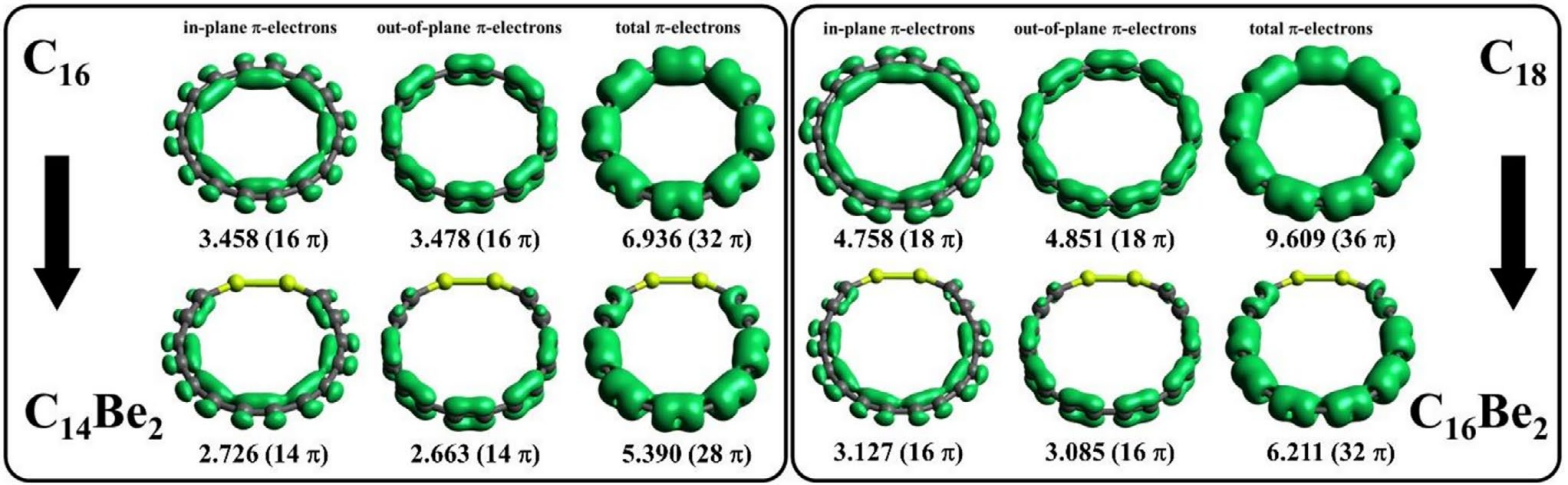
π‐EDDB plots for C_16_ and C_18_ cyclocarbons and their Be‐substituted derivatives, along with corresponding numbers of delocalized electrons.

The C_16_ and C_18_ molecules were considered the smallest representatives of antiaromatic/aromatic cyclo[n]carbons, with the most pronounced aromatic destabilization/stabilization energies. These systems contain 32 and 36 π‐electrons, distributed among 8 or 9 doubly occupied in‐plane and out‐of‐plane orbitals. The EDDB index allows for the analysis of these electrons both taken together and separately. Figure 4 presents the partition of the π‐EDDB function, showing that only about 22% and 27% of the total population of π‐electrons is delocalized in the C_16_ and C_18_ rings, respectively. Replacing an acetylenic unit with a —Be—Be— fragment disrupts electron density, as reflected in the breaking of the π‐EDDB function in this region. Quantitatively, this replacement results in a decrease in normalized π‐EDDB values, with a more pronounced decrease for the formally aromatic cyclocarbons compared to antiaromatic ones. Notably, in‐plane and out‐of‐plane π‐electrons contribute almost equally to the total π‐electron delocalization.

A similar EDDB analysis was performed for all other cyclo[n]carbons. The partition of the π‐EDDB function for other cyclo[n]carbons and their Be‐derivatives is shown in Figures S5 and S6. In these systems, about 23%–24% of the total π‐electron population participates in delocalization. Similar to C_16_ and C_18_, replacing the acetylenic unit with the —Be—Be— fragment in other cyclocarbons also leads to a decrease in π‐EDDB values, although the difference compared to the unsubstituted systems is smaller (Table 3, Table S3).

**TABLE 3:** Normalized π‐EDDB and AV1245 (multiplied by 1000) values for the studied cyclo[n]carbons and their Be‐derivatives, along with reference systems: cyclobutadiene (CBD), benzene (BNZ), and cyclooctatetraene (COT).

System	π‐EDDB^Norm [^ ^a^ ^]^			AV1245	System	π‐EDDB^Norm [^ ^a^ ^]^			AV1245
	In‐plane	Out‐of‐plane	Total			In‐plane	Out‐of‐plane	Total	
C_16_	0.216	0.217	0.217	2.208	C_14_Be_2_	0.195	0.190	0.193	2.162
C_18_	0.264	0.270	0.267	4.572	C_16_Be_2_	0.195	0.193	0.194	2.193
C_20_	0.225	0.227	0.226	2.804	C_18_Be_2_	0.199	0.198	0.199	2.352
C_22_	0.242	0.244	0.243	3.733	C_20_Be_2_	0.201	0.201	0.201	2.393
C_24_	0.229	0.230	0.230	3.063	C_22_Be_2_	0.204	0.204	0.204	2.487
C_26_	0.236	0.237	0.237	3.446	C_24_Be_2_	0.206	0.206	0.206	2.527
C_28_	0.231	0.232	0.232	3.172	C_26_Be_2_	0.209	0.209	0.209	2.587
C_30_	0.236	0.237	0.236	3.333	C_28_Be_2_	0.211	0.211	0.211	2.623
CBD	n/a	0.007	0.007	n/a					
BNZ		0.888	0.888	10.487					
COT	n/a		0.101	−0.521					

^a^ π‐EDDB^Norm^ = π‐EDDB/nπ, where nπ is the number of π‐electrons in the system.

**FIGURE 5:**
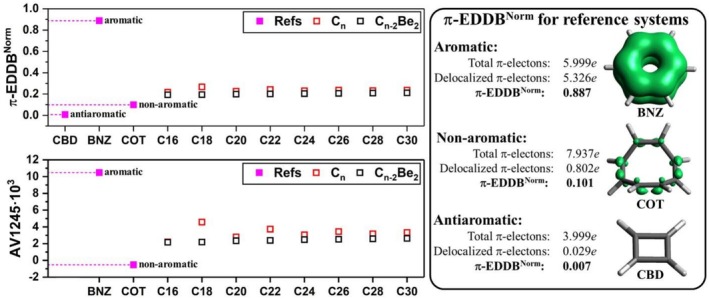
The dependence of normalized π‐EDDB and AV1245 indices on cyclo[n]carbon size, along with π‐EDDB plots and numbers of delocalized electrons for the reference systems: cyclobutadiene (CBD), benzene (BNZ), and cyclooctatetraene (COT).

To make the results more informative, we compared the π‐EDDB values of cyclo[n]carbons with those of well‐known aromatic (benzene), antiaromatic (cyclobutadiene), and non‐aromatic (cyclooctatetraene) systems. To ensure a direct comparison across these systems, we used normalized EDDB values (π‐EDDB^Norm^). As shown in Table 3 and Figure 5, the EDDB characteristics of aromatic cyclo[n]carbons differ significantly from the value of benzene, where 89% of π‐electrons are delocalized. Similarly, the π‐electron delocalization in antiaromatic cyclo[n]carbons is far from that seen in cyclobutadiene. Overall, the π‐EDDB values of cyclo[n]carbons suggest their intermediate nature, with greater resemblance to non‐aromatic cyclooctatetraene.

In addition to the EDDB index, the AV1245 electronic index [54] was computed for the cyclo[n]carbons. This index measures the average bond delocalization in positions 1–2 and 4–5 and is defined for rings with more than six atoms, making it suitable for evaluating the aromaticity of large cyclic systems. Unlike some other indices, AV1245 does not rely on reference values, is not affected by numerical precision errors, and imposes no restrictions on atom types, molecular geometry, or the level of theory used. Usually, AV1245 shows large positive values for aromatic molecules (e.g., 10.5 for benzene) and smaller positive or even negative values for antiaromatic and non‐aromatic molecules. For the studied cyclo[n]carbons, AV1245 values range from 2.2 to 4.6, significantly lower than for benzene but higher than for non‐aromatic cyclooctatetraene (Figure 5). It should be mentioned that AV1245 shows larger delocalization in cyclo[n]carbons with *n* = 4k + 2 than in cyclo[n]carbons with *n* = 4k, although this difference diminishes as the ring size increases.

Recently, Eder et al. reported the synthesis and electrochemical properties of the paracyclophanetetraene (PCT) macrocycle, which is constructed from four alternating phenyl rings and vinylene units [55]. In its neutral state, the perimeter of the macrocycle contains 24 π‐electrons, making it formally antiaromatic. The visualization of chemical shielding tensors (VIST) method applied to the PCT showed only weak global antiaromaticity [37, 56]. In contrast, its doubly reduced form, PCT^2−^ with 26 π‐electrons, demonstrates enhanced stability due to strong global aromaticity [52, 53]. Moreover, the dianion of C_12_ was studied by Yang and Cederbaum [57] highlighting its somewhat unexpected electronic structure properties.

Considering these works and taking into account that both C_18_ and C_16_ cyclocarbons can be relatively easily and reversibly reduced to their corresponding dianions, we were interested in studying the properties of the doubly reduced cyclocarbons investigated in this work. It is important to highlight that converting C_16_ and C_18_ to C_16_^2−^ and C_18_^2−^ should invert their aromatic nature. The adiabatic electron affinity analysis performed in the first part of this study revealed a remarkable difference between C_16_ and C_18_. In particular, the dianion species can exist either as a singlet, where both additional electrons occupy the same orbital, or as a triplet, where the electrons are distributed across two different orbitals. The calculations showed that for C_16_^2−^, the triplet state is more stable, whereas for C_18_^2−^, the singlet state is energetically favored (Table S1).

**FIGURE 6:**
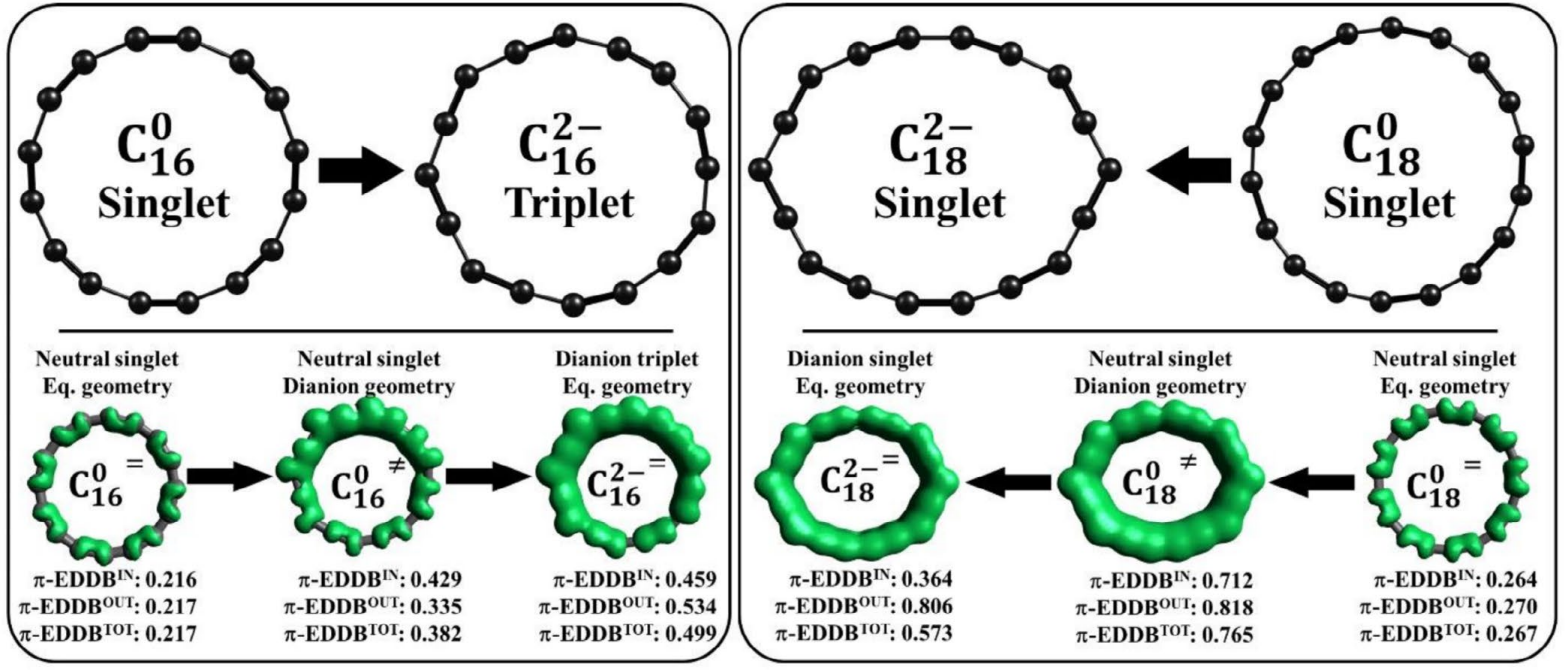
Structures, normalized π‐EDDB values, and π‐EDDB plots for C_16_ (left panel) and C_18_ (right panel) cyclocarbons in their neutral and dianionic states. Equal sign (=) corresponds to the equilibrium geometry, while not equal sign (≠) represents nonequilibrium geometry.

As shown in Figure 6, studied dianions, in both singlet and triplet states, exhibit significant distortions compared to the highly symmetrical neutral cyclocarbons. For C_18_^2−^, the geometric changes in the singlet state are especially pronounced, with the ring showing strong elongation along the axis passing through diametrically opposite carbon atoms.

Analysis of the π‐EDDB and AV1245 descriptors revealed interesting features of the studied cyclo[n]carbons (Table 4, Figure 6). The reduction of both cyclocarbons resulted in an increase in their aromaticity. This increase was expected for C_16_, as the system with 4n π‐electrons gains additional electrons, making it no longer antiaromatic. However, the increase in aromaticity for C_18_ with 4n + 2 π‐electrons was completely unexpected.

**TABLE 4:** Normalized π‐EDDB^[^ ^a^ ^]^ and AV1245 (multiplied by 1000) values for the studied cyclo[n]carbons in equilibrium neutral and dianionic states, as well as in the neutral state with geometry of the corresponding dianion.

System	Neutral singlet, equilibrium geometry		Neutral singlet, dianion geometry		Dianion, equilibrium geometry		
	π‐EDDB^Norm^	AV1245	π‐EDDB^Norm^	AV1245	State	π‐EDDB^Norm^	AV1245
C_16_	0.217	2.208	0.382	4.472	Triplet	0.499	3.783
C_18_	0.267	4.572	0.765	8.224	Singlet	0.573	5.954
C_20_	0.226	2.804	0.331	3.927	Triplet	0.459	4.298
C_22_	0.243	3.733	0.493	6.209	Singlet	0.443	5.229
C_24_	0.230	3.063	0.339	4.274	Triplet	0.452	4.767
C_26_	0.237	3.446	0.417	5.756	Triplet	0.441	5.219
C_28_	0.232	3.172	0.350	4.577	Triplet	0.447	5.084
C_30_	0.236	3.333	0.392	5.393	Triplet	0.438	5.286

^a^ The EDDB values were normalized by the total number of π‐electrons: π‐EDDB^Norm^ = π‐EDDB/nπ.

To identify the factors contributing to the increased aromaticity observed in C_16_ and C_18_, we independently examined the effects of electronic and structural distortions. Specifically, we analyzed equilibrium geometries of the neutral and dianionic forms, as well as the neutral structures constrained to the dianionic geometries. The change in aromaticity between neutral molecules in their equilibrium geometries and those constrained to the dianionic geometry captures the effect of structural distortions. In turn, the difference in aromaticity between the neutral singlet and corresponding dianionic states, both considered in the same dianionic geometry, reflects the electronic influence of reduction. For each structure, the π‐EDDB index was decomposed into in‐plane and out‐of‐plane contributions, allowing us to precisely assess how structural and electronic factors contribute to aromaticity changes.

As shown in Figure 6, the transition from the neutral to the dianionic geometry leads to an increase in aromaticity for both C_16_ and C_18_ cyclocarbons. For C_16_, this increase is moderate, with the π‐EDDB^TOT^ value rising from 0.217 to 0.382. However, in C_18_, the aromaticity change is much more pronounced, as indicated by the π‐EDDB^TOT^ value increasing from 0.267 to 0.765. This rise in aromaticity most likely correlates with equalization of bond lengths within the molecule. To quantify this effect, we calculated the bond length alternation (BLA) index, which measures the average difference between adjacent bond lengths. As expected, a significant reduction in BLA was observed for both cyclocarbons. In particular, the BLA index for C_16_ decreased from 0.150 to 0.061 Å, while for C_18_, it dropped from 0.123 to 0.065 Å. This substantial bond length equalization in the dianionic geometry supports the observed increase in electron delocalization and enhanced aromaticity for both systems. This observation is in line with our previous work, where we showed that structural distortions such as uniaxial tension and radial expansion/contraction can modify the (anti)aromatic properties of the C_16_ and C_18_ cyclocarbons [58]. The results are also consistent with Ref. [57] highlighting a similar kink and bond‐length equalization in C_12_^2−^.

The reduction process, along with the associated changes in electronic states, also significantly impacts the aromaticity of the systems. For C_16_, the addition of two extra electrons further enhances aromaticity, increasing the π‐EDDB^TOT^ value from 0.382 to 0.499. It is important to note that C_16_^2−^ adopts a triplet state, where one of the additional electrons occupies an in‐plane π‐orbital, while the other resides in an out‐of‐plane π‐orbital (see Figure S7). This configuration transforms the originally antiaromatic neutral C_16_ molecule, which follows a 4n electron rule in both the in‐plane and out‐of‐plane π‐systems, into the C_16_^2−^ dianion, which now has 4n + 1 electrons in each π‐electronic system, thereby promoting aromaticity. Thus, in C_16_, both structural and electronic factors act together to promote the aromaticity of C_16_^2−^.

In contrast to C_16_, the reduction of C_18_^≠^ to C_18_^2−^ results in a decrease in aromaticity, as indicated by a drop in the π‐EDDB^TOT^ value from 0.765 to 0.573. The C_18_^2−^ dianion adopts a singlet electronic configuration, where both added electrons occupy the same in‐plane orbital (see Figure S7). This electron addition transforms the in‐plane electronic system from an aromatic configuration with 4n + 2 electrons to an antiaromatic configuration with 4n electrons. As a result, the in‐plane π‐EDDB^IN^ value decreases significantly, from 0.712 to 0.364. Meanwhile, the out‐of‐plane electronic system remains mostly unaffected by the reduction process, with the π‐EDDB^OUT^ values showing only a slight decrease, from 0.818 to 0.806 (Figure 6). Thus, in C_18_, the effects of geometric distortion and electronic factors oppose each other: while electronic changes reduce aromaticity, the influence of bond length equalization predominates, ultimately resulting in the aromaticity increase of C_18_^2−^.

As shown in Table 4, an increase in aromaticity upon reduction is observed not only for C_16_ and C_18_ cyclocarbons but also across all the cyclo[n]carbons studied. It is also noteworthy that a similar trend is captured by the AV1245 index. Like the π‐EDDB descriptor, AV1245 shows a significant increase in aromaticity due to geometric distortion, followed by further increases or decreases depending on the inherent aromatic character of each cyclo[n]carbon. Thus, the analysis of cyclocarbon dianions served not only to verify the aromaticity of the neutral systems but also to test the consistency and responsiveness of aromaticity descriptors and to provide complementary insight into how π‐electron delocalization evolves upon electron addition.

It is important to note that the aromatic nature and the most stable electronic configuration of dianions alternate only in smaller cyclo[n]carbons (from C_16_ to C_22_). However, starting at C_24_, the triplet state consistently becomes the most stable electronic configuration for the dianions, and differences in electronic aromaticity indices between cyclo[n]carbons with *n* = 4k + 2 and 4k become very small, particularly within 3% for π‐EDDB and 11% for AV1245. The similar tendency was found by the ASE analysis: alternation in aromatic character is observed only in the smaller members of the cyclo[n]carbon family. This consistency between π‐EDDB, AV1245 and ASE descriptors reinforces the reliability of these metrics in assessing aromaticity across different cyclo[n]carbons.

Summing up, the analysis of factors influencing the aromaticity of the studied cyclo[n]carbons and their dianions leads to the following conclusions:

- The reduction of cyclo[n]carbons to their corresponding dianions induces geometric distortion, which equalizes the C—C bond lengths and enhances the aromaticity of the systems.
- The aromaticity changes resulting from the addition of two electrons align well with the predictions of aromaticity and antiaromaticity according to Hückel's rule.
- For antiaromatic cyclo[n]carbons, such as C_16_ and C_20_, both structural and electronic factors act synergistically to increase aromaticity of dianions. In contrast, for aromatic cyclo[n]carbons, such as C_18_ and C_22_, structural and electronic factors oppose each other. However, the increase in aromaticity due to geometric distortion is greater than the opposing effect of electronic factors. As a result, the transition from the neutral form to the dianionic form consistently leads to an overall increase in aromaticity for each system.

## 3 Conclusions

While even‐numbered cyclo[n]carbons have traditionally been classified as doubly aromatic (*n* = 4k + 2) or doubly antiaromatic (*n* = 4k), our computational results indicate that these systems are more accurately described as at most weakly aromatic or antiaromatic and that any signature of (anti)aromaticity fades out at about *n* = 24.

Adiabatic electron affinities show similar trends across both classes, suggesting minimal influence of (anti)aromaticity on electronic properties. Aromatic stabilization energies, derived from both homodesmotic and disproportionation reactions, are consistently low, especially for molecules with *n* ≥ 24, where ASE values fall below 2 kcal/mol, indicating very low thermodynamic stabilization. EDDB analysis further confirms that less than 30% of π‐electrons participate in delocalization, with only minor differences between *n* = 4k and 4k + 2 systems that diminish with size.

Upon two‐electron reduction, all systems exhibit increased aromaticity, driven by bond length equalization and π‐electron enhanced delocalization. In cyclo[n]carbons with *n* = 4k (e.g., C_16_ and C_20_), structural and electronic effects act synergistically to increase aromaticity, while in systems with *n* = 4k + 2 (e.g., C_18_ and C_22_), they compete, but with the dominant influence of geometric distortion.

Taken together, our findings demonstrate that small neutral cyclo[n]carbons (*n* < 24) possess only weak aromatic or antiaromatic character, with low stabilization energies and moderate electron delocalization, while characteristics of bigger systems allow us to classify them as non‐aromatic. Note that the aromaticity of all studied cyclo[n]carbons becomes more pronounced in their dianionic states, driven by structural and electronic reorganization.

## Conflicts of Interest

The authors declare no conflicts of interest.

## Supporting information


**Data S1:** jcc70283‐sup‐0001‐supinfo.pdf.

## Data Availability

The data that supports the findings of this study are available in the Supporting Information of this article. It contains: detailed computational methodology; calculated adiabatic electron affinities; π‐EDDB aromaticity indices and corresponding π‐EDDB plots; graphical representations of (anti)aromatic cyclo[n]carbons and their beryllium derivatives; Cartesian coordinates of all optimized structures.
