# Supplementary material for: The (Anti)aromatic Properties of Cyclo[n]Carbons: Myth or Reality?
Source: J Comput Chem. 2025 Nov 29;46(31):e70283. doi: 10.1002/jcc.70283 (PMC12663756; doi:10.1002/jcc.70283)
Supplement: Supplementary file 1 — Data S1: jcc70283‐sup‐0001‐supinfo.pdf. [file JCC-46-0-s001.pdf]

Supporting information for:

The (anti)aromatic properties of cyclo[n]carbons: myth or reality?

O. A. Stasyuk,*[a] G. George,[a] C. Curutchet,[b,c] F. Plasser,[d] and A. J. Stasyuk*[b,c,e]

a. Institut de Química Computacional i Catàlisi and Departament de Química, Universitat de Girona, C/ Maria Aurèlia Capmany 69, 17003 Girona, Catalonia, Spain

b. Departament de Farmàcia i Tecnologia Farmacèutica, i Fisicoquímica, Facultat de Farmàcia i Ciències de l'Alimentació, Universitat de Barcelona (UB), Av. Joan XXIII 27-31, 08028, Barcelona, Spain

c. Institut de Química Teòrica i Computacional (IQTCUB), Universitat de Barcelona (UB), Barcelona, Spain

d. Department of Chemistry, Loughborough University, Loughborough LE11 3TU, U.K.

e. Faculty of Chemistry, University of Warsaw, Pasteura 1, 02-093 Warsaw, Poland

*Corresponding authors. e-mail: anton.stasuk@gmail.com; o.a.stasuk@gmail.com

Abstract

Recent advances in on-surface chemistry have enabled the synthesis and structural characterization of even-numbered cyclo[n]carbons, traditionally classified as either doubly aromatic (n = 4k+2) or doubly antiaromatic (n = 4k) based on their in-plane and out-of-plane π-electron circuits. However, recent studies have increasingly questioned this classification, suggesting instead that these molecules are more accurately described as non-aromatic. In this work, we computationally examine the electron affinities and (anti)aromatic character of cyclo[n]carbons with n = 16-30 using energetic, structural, and electronic aromaticity descriptors. Adiabatic electron affinity (AEA) analysis reveals a high degree of uniformity across the series of both nominally aromatic and antiaromatic members. Aromatic stabilization energy (ASE) values, derived from homodesmotic and disproportionation reactions, indicate slight destabilization only for C_{16} and C_{20}, and low stabilization for the remaining systems. In particular, ASE is less than 2 kcal/mol for cyclo[n]carbons with n ≥ 24. This suggests that neither aromatic nor antiaromatic character significantly contributes to the thermodynamic stability of larger cyclocarbons. EDDB analysis further supports this conclusion, with only about 22-27% of π-electrons participating in delocalization. While delocalization is slightly greater in cyclo[n]carbons with n = 4k+2, the difference diminishes with increasing size. Upon two-electron reduction to the dianionic state, all cyclo[n]carbons exhibit bond length equalization and increased delocalization. These results suggest that only small cyclo[n]carbons (n < 24) can be classified as weakly (anti)aromatic, while larger cyclo[n]carbons (n ≥ 24) are more appropriately classified as non-aromatic systems. The aromaticity of all considered cyclocarbons becomes more pronounced in corresponding dianionic forms due to cooperative structural and electronic effects. Thus, this work provides a unified framework for interpreting and predicting the electronic behavior of cyclocarbons.

Table of Contents

1.	Computational methodology	S3
2.	**Figure S1.** Adiabatic electron affinities for aromatic and antiaromatic cyclo[n]carbons in the gas phase and within CPCM solvation model.	S4
3.	**Figure S2.** Graphical representation for aromatic cyclo[n]carbon and their beryllium derivatives.	S4
4.	**Figure S3.** Graphical representation for antiaromatic cyclo[n]carbon and their beryllium derivatives.	S5
5.	**Figure S4.** HOMOs for $C_{n-2}Be_2$ cyclo[n]carbon derivatives.	S5
6.	**Figure S5.** π-EDDB plots for aromatic C_{22}, C_{26}, and C_{30} cyclocarbons.	S6
7.	**Figure S6.** π-EDDB plots for aromatic C_{20}, C_{24}, and C_{28} cyclocarbons.	S6
8.	**Figure S7.** Selected HOMO and LUMO orbitals for neutral C_{16}, C_{18} and corresponding dianions.	S7
9.	**Table S1.** Gibbs free energies and calculated first (AEA1) and second (AEA2) adiabatic electron affinities.	S8
10.	**Table S2.** Two sample t-test for AEA1 and AEA2 values for studied aromatic and antiaromatic cyclo[n]carbons.	S8
11.	**Table S3.** π-EDDB aromaticity indices for the studied cyclo[n]carbons.	S9
12.	Cartesian coordinates	S10
	References	S19

Computational Methodology

Quantum-chemical calculations

Geometry optimizations were performed employing the DFT ωB97XD [1] long-range corrected hybrid functional with built-in empirical atomic-pairwise dispersion correction in conjunction with the ma-def2-TZVPP basis set [2,3], which includes polarization and diffuse functions. Normal mode vibrational frequencies were calculated in each case at the same level of theory. Molecular structures and frontier molecular orbitals were visualized by Chemcraft 1.8 program.[4]

Aromaticity

EDDB

Electron density of delocalized bonds (EDDB) is a part of the original method of one-electron density (ED) decomposition into 'layers' representing different levels of electron delocalization [5]. In the basis of natural atomic orbitals (NAO), or any other representation of well-localized orthonormalized atomic orbitals, the spinless global electron density of delocalized bonds function, $\mathrm{EDDB_G}(r)$, for a single-determinant molecular wavefunction is defined as follows: [6,7]

$$\mathrm{EDDB_G}(r) = \sum_{\mu,\nu} \chi_\mu^\dagger(r) \mathcal{D}_{\mu,\nu}^{\Omega_\mathrm{G}} \chi_\nu(r) \tag{1}$$

where

$$\mathscr{D}^{\Omega_G} = 2 \sum_{\sigma=\alpha,\beta} \mathbf{P}^\sigma \left[\sum_{a,b}^{\Omega_G} \mathcal{C}_{a,b}^\sigma \mathcal{E}_{a,b}^{\Omega_G,\sigma} (\lambda_{a,b}^\sigma)^2 \mathcal{C}_{a,b}^{\sigma\dagger} \right] \mathbf{P}^\sigma \tag{2}$$

In the above equation, P^σ ($\sigma = \alpha,\beta$) stands for the σ spin-resolved charge and bond-order (CBO) matrix, $\mathcal{C}_{a,b}^\sigma$ is the matrix of linear coefficients of the appropriately orthogonalized σ spin-resolved two-center bond-order orbitals (2cBO) of the chemical bond X_a–X_b (obtained by diagonalization of the appropriate off-diagonal blocks of the CBO matrix), $\lambda_{a,b}^\sigma$ represents the diagonal matrix collecting the corresponding 2cBO eigenvalues (occupation numbers), $\mathcal{E}_{a,b}^{\Omega G.\sigma}$ is a diagonal matrix of the σ-spin bond-conjugation factors, and for an n-atomic molecular system, Ω_G represents the set of all n(n-1)/2 possible atomic pairs (regardless of whether the atoms are formally bonded or not). The definition of the key matrix $\mathcal{E}_{a,b}^{\Omega G.\sigma}$ is based on the bond-orbital projection (BOP) criterion developed by one of the authors, which relies on sophisticated orbital projection cascades involving 2cBOs, their 3-center counterparts (3cBO), and canonical MOs. According to BOP, for a typical well-localized (Lewis-like) bond X_a–X_b, all diagonal elements of the $\mathcal{E}_{a,b}^{\Omega G.\sigma}$ matrix are close to zero, which means that the 2cBOs associated with this bond do not form effectively linear combinations with 2cBOs of all other bonds in a molecule. On the other hand, when the X_a–X_b bond is effectively conjugated with any other adjacent bond in the system, the $\mathcal{E}_{a,b}^{\Omega G.\sigma}$ matrix has at least one element on its diagonal that approaches 1 (for systems with double and higher multifaceted

aromaticity, the number of non-zero diagonal elements is equal to the number of delocalization 'channels').

The trace of such defined $\mathcal{D}^{\Omega G}$ matrix can be straightforwardly interpreted as the population of electrons delocalized through the system of all conjugated bonds in a molecule, and as such, it can be used as a 'measure' of *global* aromaticity [6, 7]. However, one of the most distinctive features of the BOP technique is that one can easily restrict the set bonds/atomic pairs in Ω_G giving rise to a series of different variants of *global* and *local* EDDB functions.

AV1245

AV1245 index evaluates the average delocalization along the ring and corresponds to the average value of the four-atom multicenter index (MCI) between relative positions 1–2 and 4–5 built from each five-atom fragment along the perimeter of the ring [8]. AV1245 does not rely on reference values, and it does not present any limitation on the nature of atoms, the molecular geometry or the level of calculation. Aromatic molecules are typically characterized by larger AV1245 values than antiaromatic and nonaromatic counterparts. However, the difference between aromatic and antiaromatic macrocycles becomes smaller as the size of the macrocycle increases.

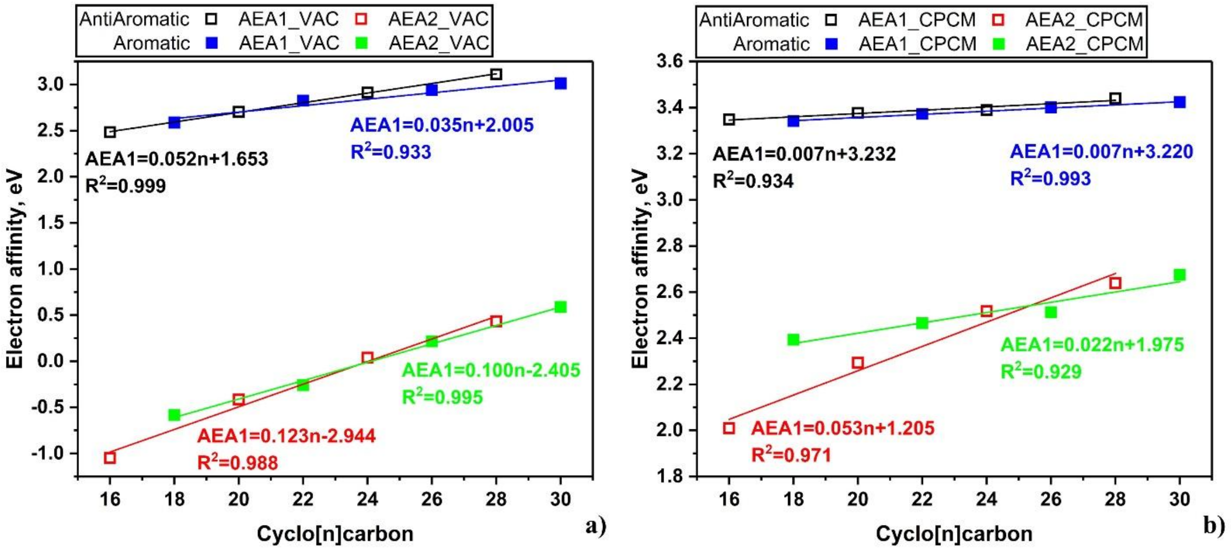

Figure S1. Adiabatic electron affinities for aromatic and antiaromatic cyclo[n]carbons in the gas phase (a) and within CPCM solvation model, ε = 3.0 (b).

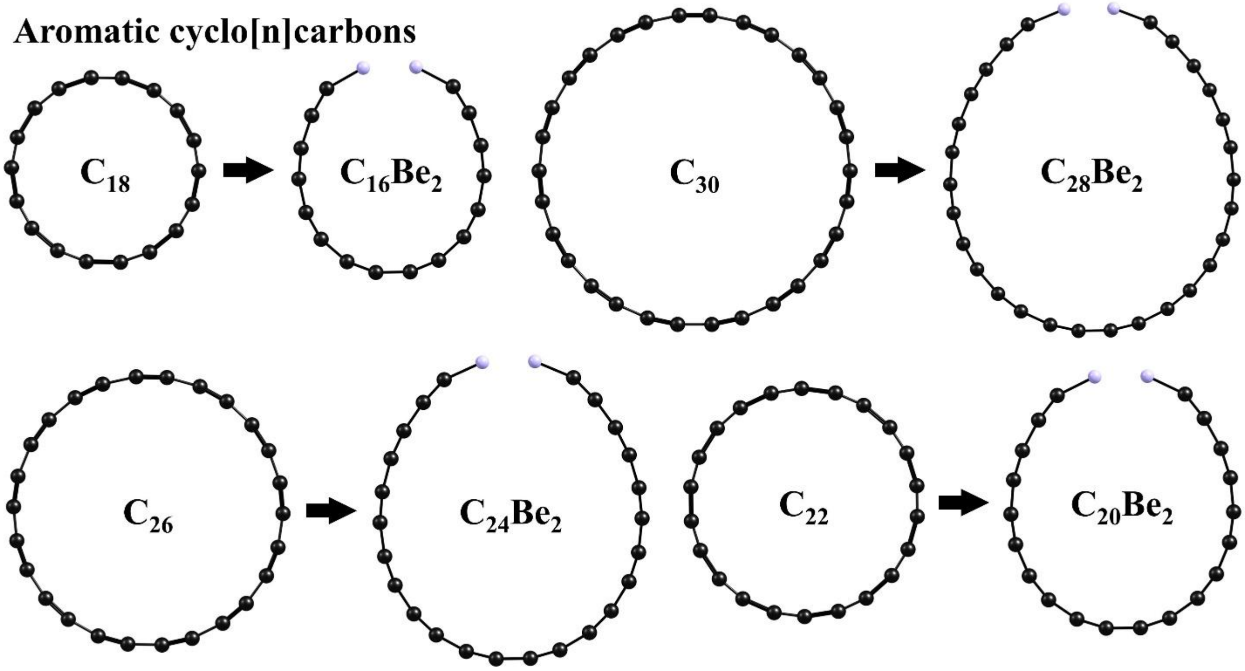

Figure S2. Graphical representation for aromatic cyclo[n]carbons and their beryllium derivatives.

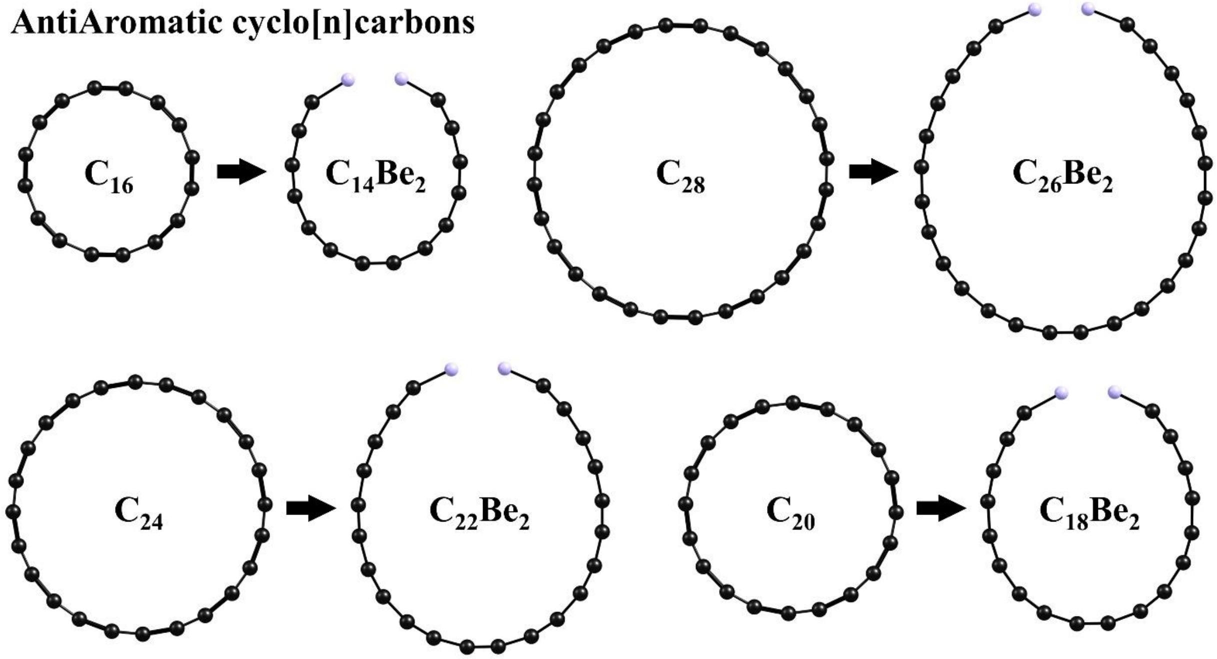

Figure S3. Graphical representation for antiaromatic cyclo[n]carbons and their beryllium derivatives.

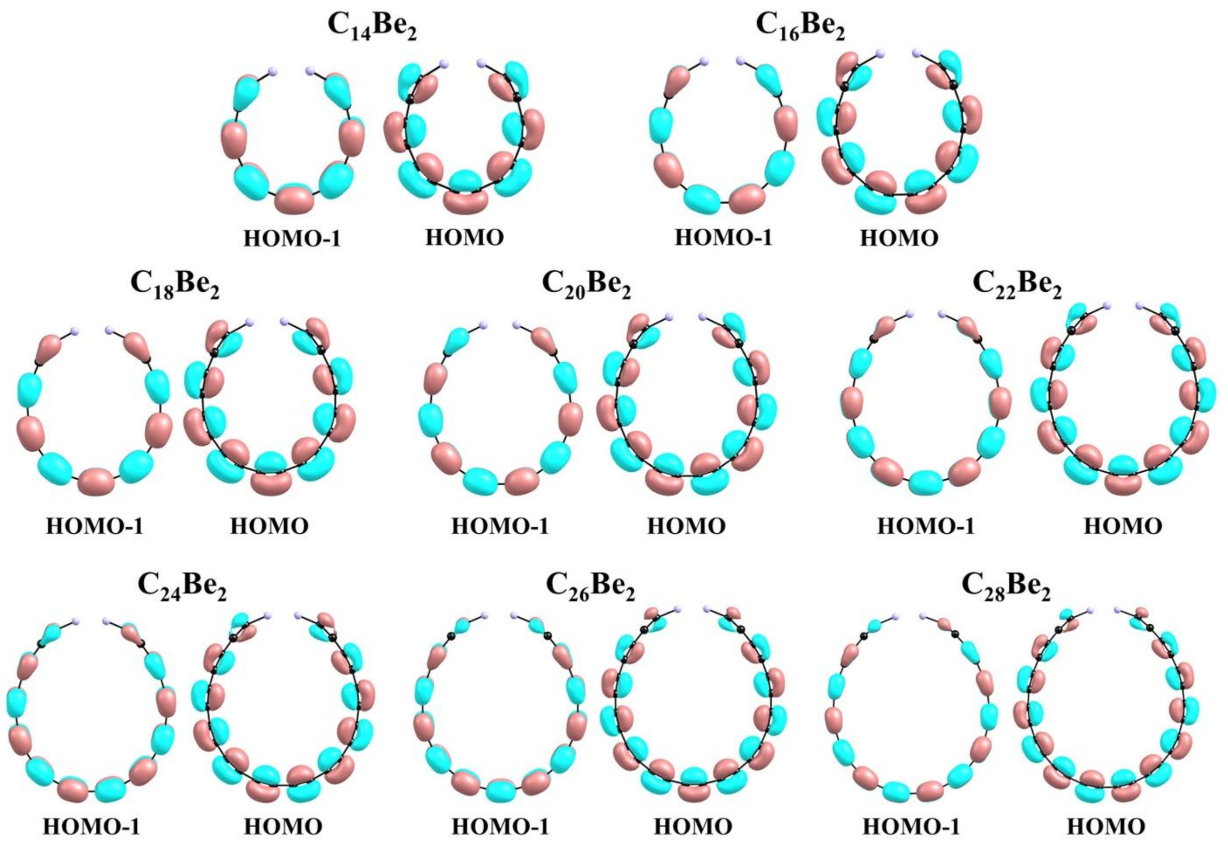

Figure S4. HOMOs for $C_{n-2}Be_2$ cyclo[n]carbon derivatives.

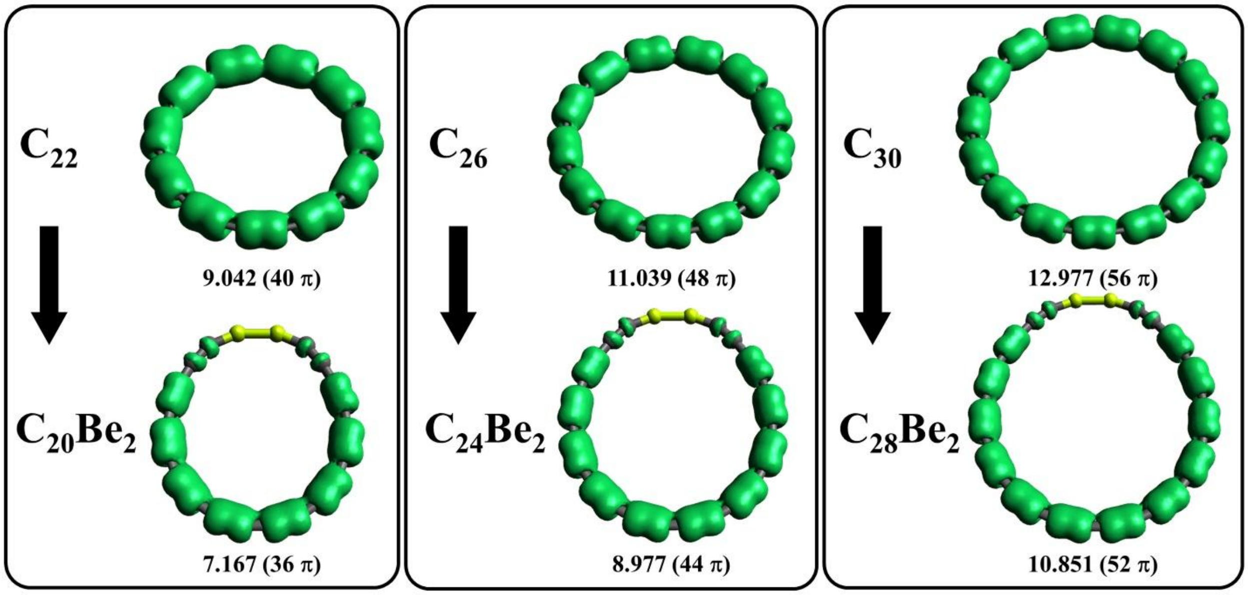

Figure S5. π-EDDB plots for aromatic C_{22}, C_{26}, and C_{30} cyclocarbons and their Be-derivatives.

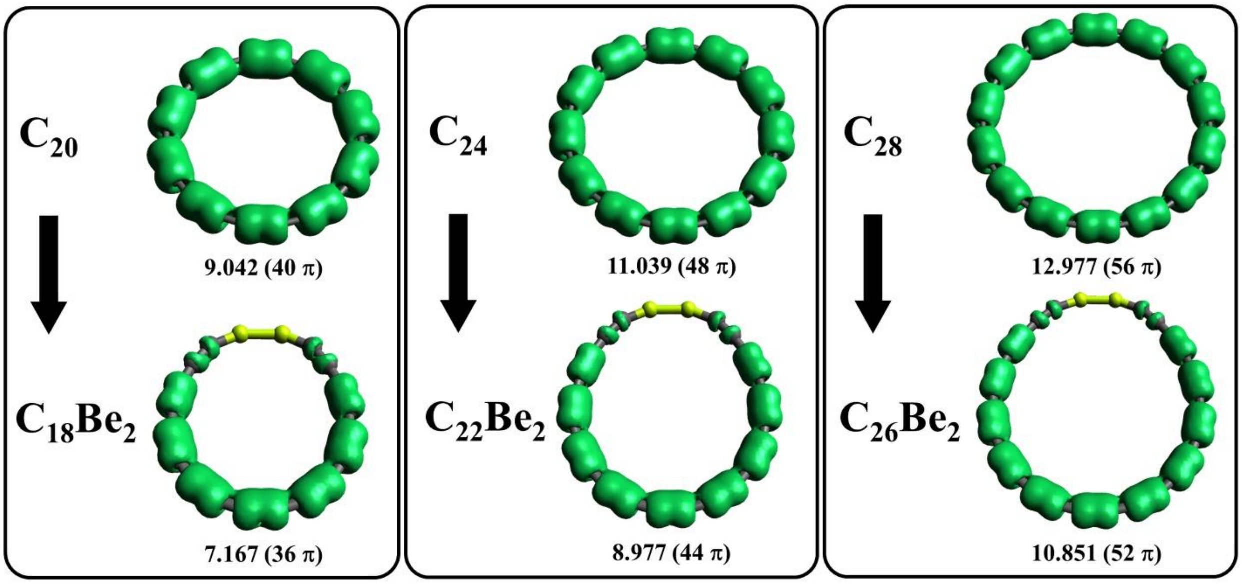

Figure S6. π-EDDB plots for antiaromatic C_{20}, C_{24}, and C_{28} cyclocarbons and their Be-derivatives.

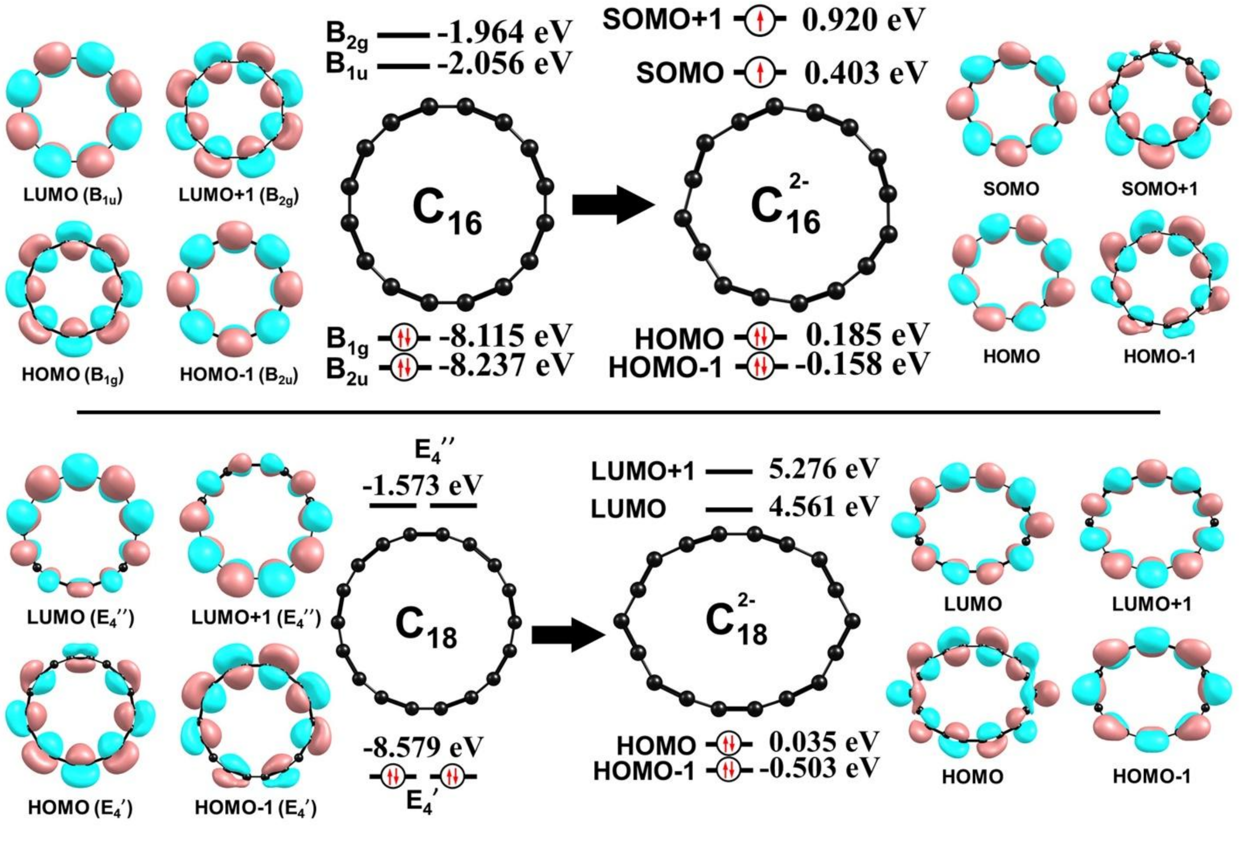

Figure S7. Selected HOMO and LUMO orbitals for neutral C_{16} and C_{18}, as well as their C_{16}^{2-} and C_{18}^{2-} dianions.

Table S1. Gibbs free energies[a] and calculated first (AEA1) and second (AEA2) adiabatic electron affinities in the gas phase for cyclo[n]carbons of interest.

System	Neutral singlet	Mono anion	Dianion singlet	Dianion triplet	AEA1[b]	AEA2[b]
C_{16}	-609.038704	-609.130023	-609.088902	-609.091467	2.485	-1.049
C_{18}	-685.206493	-685.301577	-685.280102	-685.269489	2.587	-0.584
C_{20}	-761.348725	-761.448048	-761.418087	-761.432753	2.703	-0.416
C_{22}	-837.503576	-837.607376	-837.597828	-837.596516	2.825	-0.260
C_{24}	-913.647397	-913.754458	-913.751591	-913.755890	2.913	0.039
C_{26}	-989.797531	-989.905532	-989.908573	-989.913469	2.939	0.216
C_{28}	-1065.940610	-1066.054966	-1066.062499	-1066.070895	3.112	0.433
C_{30}	-1142.090934	-1142.201673	-1142.215163	-1142.223259	3.013	0.587

[a] Preferential electronic state for dianions is highlighted in grey; [b] $AEA1 = \Delta G^{eq}_{C_n^0} - \Delta G^{eq}_{C_n^{1-}}; AEA2 = \Delta G^{eq}_{C_n^{1-}} - \Delta G^{eq}_{C_n^{2-}}$

Table S2. Two sample t-test for AEA1 and AEA2 values for aromatic and antiaromatic cyclo[n]carbons.

	Two sample t-test							
	gas phase							
	AEA1				AEA2			
1	C_{16}	2.485	C_{18}	2.587	C_{16}	-1.049	C_{18}	-0.584
2	C_{20}	2.703	C_{22}	2.825	C_{20}	-0.416	C_{22}	-0.260
3	C_{24}	2.913	C_{26}	2.939	C_{24}	0.039	C_{26}	0.216
4	C_{28}	3.112	C_{30}	3.013	C_{28}	0.433	C_{30}	0.587
Mean	2.803		2.841		-0.248		-0.010	
Variance	0.0547		0.0260		0.3042		0.2001	
Standard Deviation	0.2339		0.1612		1.2169		0.8002	
t-value/ critical value at $p < 0.001$	-0.2665/5.959				-0.5808/5.959			
Decision	No significant difference revealed				No significant difference revealed			
	CPCM							
	AEA1				AEA2			
1	C_{16}	3.349	C_{18}	3.341	C_{16}	2.010	C_{18}	2.393
2	C_{20}	3.377	C_{22}	3.373	C_{20}	2.293	C_{22}	2.465
3	C_{24}	3.389	C_{26}	3.401	C_{24}	2.517	C_{26}	2.512
4	C_{28}	3.440	C_{30}	3.423	C_{28}	2.638	C_{30}	2.675
Mean	3.389		3.385		2.3645		2.5113	
Variance	0.0011		0.0010		0.0572		0.0107	
Standard Deviation	0.0332		0.0316		0.2392		0.1034	
t-value/ critical value at $p < 0.001$	0.1855/5.959				-1.1261/5.959			
Decision	No significant difference revealed				No significant difference revealed			

Table S3. π-EDDB aromaticity indices for the studied cyclo[n]carbons.

	π-EDDB					
	in-plane / out-of-plane			Total (in and out)		
	deloc. elect	num elect	π-EDDB^{Norm}	deloc. elect	num elect	π-EDDB^{Norm}
	Cyclo[n]carbons					
C_{16}	3.457/3.478	16.000/16.000	0.216/0.217	6.936	32.000	0.217
C_{18}	4.758/4.851	18.000/18.000	0.264/0.270	9.609	36.000	0.267
C_{20}	4.494/4.548	20.000/20.000	0.225/0.227	9.042	40.000	0.226
C_{22}	5.321/5.369	22.000/22.000	0.242/0.244	10.690	44.000	0.243
C_{24}	5.507/5.531	24.000/24.000	0.230/0.231	11.039	48.000	0.230
C_{26}	6.132/6.175	26.000/26.000	0.236/0.238	12.307	52.000	0.237
C_{28}	6.477/6.499	28.000/28.000	0.231/0.232	12.977	56.000	0.232
C_{30}	7.075/7.103	30.000/30.000	0.236/0.237	14.178	60.000	0.236
	Be-derivatives					
$C_{14}Be_2$	2.726/2.663	14.000/14.000	0.195/0.190	5.390	28.000	0.193
$C_{16}Be_2$	3.127/3.085	16.000/16.000	0.195/0.193	6.211	32.000	0.194
$C_{18}Be_2$	3.596/3.572	18.000/18.000	0.200/0.198	7.167	36.000	0.199
$C_{20}Be_2$	4.027/4.016	20.000/20.000	0.201/0.201	8.043	40.000	0.201
$C_{22}Be_2$	4.485/4.492	22.000/22.000	0.204/0.0204	8.977	44.000	0.204
$C_{24}Be_2$	4.946/4.950	24.000/24.000	0.206/0.206	9.896	48.000	0.206
$C_{26}Be_2$	5.427/5.424	26.000/26.000	0.209/0.209	10.851	52.000	0.209
$C_{28}Be_2$	5.911/5.917	28.000/28.000	0.211/0.211	11.828	56.000	0.211

π-EDDB^{Norm} = π-EDDB/nπ

Cartesian coordinates

Neutral cyclo[n]carbons

C16

Gas-phase. wB97XD/ma-def2-TZVPP

Atom	X	Y	Z
6	0.387419000	3.277805000	0.000208000
6	1.545414000	2.916870000	-0.000011000
6	2.592028000	2.043955000	-0.000180000
6	3.155616000	0.969900000	-0.000222000
6	2.044088000	-2.591850000	-0.000082000
6	-0.387412000	-3.277766000	0.000065000
6	-1.545415000	-2.916860000	-0.000223000
6	-2.592040000	-2.043957000	-0.000025000
6	-3.155631000	-0.969903000	0.000278000
6	-0.969898000	3.155200000	0.000041000
6	-2.044061000	2.591818000	-0.000067000
6	0.969907000	-3.155198000	0.000185000
6	-2.917154000	1.545353000	0.000064000
6	3.278276000	-0.387425000	-0.000102000
6	-3.278299000	0.387422000	0.000168000
6	2.917160000	-1.545365000	-0.000098000

C18

Gas-phase. wB97XD/ma-def2-TZVPP

Atom	X	Y	Z
6	-2.231824000	2.941241000	0.000545000
6	-3.136923000	1.947715000	0.000397000
6	-3.600643000	0.818580000	0.000265000
6	-3.655380000	-0.524290000	0.000121000
6	-1.431376000	-3.403391000	-0.000142000
6	2.281603000	-2.903155000	-0.000374000
6	3.103578000	-2.000796000	-0.000531000
6	3.613960000	-0.757500000	-0.000369000
6	3.663368000	0.462134000	-0.000129000
6	0.181023000	3.687755000	0.000393000
6	1.373554000	3.427457000	0.000184000
6	-0.118361000	-3.690222000	-0.000272000
6	2.509273000	2.708820000	0.000040000
6	-3.284495000	-1.687214000	0.000111000
6	3.255235000	1.742646000	-0.000007000
6	-2.462999000	-2.750896000	-0.000230000
6	-1.150942000	3.508411000	0.000392000
6	1.091350000	-3.527295000	-0.000396000

C20

Gas-phase. wB97XD/ma-def2-TZVPP

Atom	X	Y	Z
6	0.599196000	-4.063154000	0.000011000
6	1.890482000	-3.646555000	0.000005000
6	2.874115000	-2.935782000	0.000004000
6	3.673792000	-1.839573000	-0.000003000
6	4.050679000	-0.685991000	-0.000003000
6	4.053199000	0.670845000	-0.000005000
6	2.885064000	2.924991000	0.000000000
6	3.680729000	1.825860000	-0.000004000
6	1.904103000	3.639449000	0.000011000
6	-0.599196000	4.063153000	-0.000011000
6	-1.890483000	3.646556000	-0.000005000
6	-2.874115000	2.935782000	-0.000004000
6	-3.673791000	1.839573000	0.000003000
6	-4.050678000	0.685990000	0.000003000
6	-4.053200000	-0.670846000	0.000005000
6	-3.680730000	-1.825861000	0.000004000
6	-2.885064000	-2.924990000	0.000000000
6	-0.614369000	-4.060819000	-0.000010000
6	-1.904103000	-3.639448000	-0.000011000
6	0.614369000	4.060819000	0.000010000

C22

Gas-phase. wB97XD/ma-def2-TZVPP

Atom	X	Y	Z
6	-3.456539000	-2.893334000	0.000000000
6	-4.472122000	-0.565285000	0.000000000
6	-4.460469000	0.651178000	0.000000000
6	-4.067851000	1.942258000	0.000000000
6	-3.400315000	2.959267000	0.000000000
6	-2.371995000	3.833107000	0.000000000
6	0.076910000	4.506988000	0.000000000
6	-1.260600000	4.327793000	0.000000000
6	1.279323000	4.322300000	0.000000000
6	3.413106000	2.944539000	0.000000000
6	4.463218000	0.631861000	0.000000000
6	4.450226000	-0.717534000	0.000000000
6	4.096259000	-1.881416000	0.000000000
6	3.355819000	-3.009593000	0.000000000
6	2.428828000	-3.797371000	0.000000000
6	1.195990000	-4.346136000	0.000000000
6	-0.009754000	-4.507671000	0.000000000
6	-2.445235000	-3.786809000	0.000000000
6	-1.343566000	-4.302786000	0.000000000
6	2.501402000	3.749962000	0.000000000
6	-4.104379000	-1.863666000	0.000000000
6	4.131746000	1.802348000	0.000000000

C24

Gas-phase. wB97XD/ma-def2-TZVPP

Atom	X	Y	Z
6	4.366186000	-2.264660000	0.000635000
6	3.583273000	-3.369430000	0.000172000
6	0.221787000	-4.913000000	-0.000069000
6	-1.126427000	-4.787266000	0.000053000
6	-2.264733000	-4.365894000	0.000302000
6	-3.369603000	-3.583118000	0.000044000
6	-4.709711000	-1.418403000	-0.000672000
6	-4.144550000	-2.648883000	-0.000391000
6	-4.913507000	-0.221830000	-0.000901000
6	-4.366078000	2.264628000	-0.000149000
6	-3.583184000	3.369414000	0.000333000
6	-2.648909000	4.144313000	0.000848000
6	-1.418402000	4.709414000	0.000914000
6	-0.221826000	4.913200000	0.000663000
6	1.126370000	4.787264000	0.000134000
6	2.264655000	4.365835000	-0.000416000
6	3.369494000	3.583014000	-0.000738000
6	4.709697000	1.418353000	-0.000103000
6	4.144473000	2.648804000	-0.000705000
6	-4.787569000	1.126366000	-0.000675000
6	4.787768000	-1.126431000	0.000609000
6	4.913509000	0.221784000	0.000234000
6	1.418367000	-4.709239000	-0.000077000
6	2.648920000	-4.144235000	-0.000042000

C26

Gas-phase. wB97XD/ma-def2-TZVPP

Atom	X	Y	Z
6	-5.322185000	-0.014734000	0.000362000
6	-5.180199000	-1.221353000	0.000349000
6	-3.011003000	-4.387707000	0.000306000
6	-1.937246000	-4.956151000	0.000259000
6	-0.626837000	-5.284280000	0.000130000
6	0.588094000	-5.288747000	0.000005000
6	3.993319000	-3.517707000	-0.000351000
6	2.978756000	-4.409612000	-0.000242000
6	4.687423000	-2.520562000	-0.000415000
6	5.322063000	-0.053769000	-0.000271000
6	5.163904000	1.287803000	-0.000117000
6	4.737114000	2.425304000	-0.000012000
6	3.973759000	3.539824000	-0.000075000
6	3.067263000	4.348735000	-0.000133000
6	1.873372000	4.980779000	-0.000204000
6	0.694780000	5.275768000	-0.000223000
6	-0.656081000	5.280692000	-0.000162000
6	-3.947744000	3.568829000	0.000185000
6	-3.035219000	4.370939000	0.000075000
6	5.171211000	-1.259298000	-0.000362000
6	-5.154488000	1.325688000	0.000284000
6	-4.719359000	2.460029000	0.000230000
6	-4.019061000	-3.488440000	0.000287000
6	-4.705711000	-2.486154000	0.000298000
6	1.900861000	-4.970181000	-0.000129000
6	-1.836785000	4.994306000	-0.000075000

C28

Gas-phase. wB97XD/ma-def2-TZVPP

Atom	X	Y	Z
6	-3.121469000	4.806047000	0.000000000
6	-4.161116000	3.940556000	0.000000000
6	-5.703984000	0.556157000	0.000000000
6	-5.675446000	-0.796294000	0.000000000
6	-5.380403000	-1.973697000	0.000000000
6	-4.767837000	-3.179807000	0.000000000
6	-1.811439000	-5.436890000	0.000000000
6	-2.915921000	-4.933464000	0.000000000
6	-0.486541000	-5.709995000	0.000000000
6	3.121469000	-4.806047000	0.000000000
6	4.161116000	-3.940556000	0.000000000
6	4.897773000	-2.975848000	0.000000000
6	5.458911000	-1.744966000	0.000000000
6	5.703984000	-0.556157000	0.000000000
6	5.675446000	0.796293000	0.000000000
6	5.380403000	1.973697000	0.000000000
6	4.767837000	3.179807000	0.000000000
6	1.811439000	5.436890000	0.000000000
6	2.915921000	4.933464000	0.000000000
6	0.726996000	-5.684348000	0.000000000
6	-0.726996000	5.684348000	0.000000000

C30

Gas-phase. wB97XD/ma-def2-TZVPP

Atom	X	Y	Z
6	-5.478811000	-2.763782000	0.000000000
6	-5.915804000	-1.630901000	0.000000000
6	-5.719967000	2.222014000	0.000000000
6	-5.170378000	3.304759000	0.000000000
6	-4.321576000	4.356280000	0.000000000
6	-3.379095000	5.121864000	0.000000000
6	0.345773000	6.126224000	0.000000000
6	-1.003617000	6.053364000	0.000000000
6	1.545369000	5.938183000	0.000000000
6	5.447362000	2.825105000	0.000000000
6	5.933724000	1.564303000	0.000000000
6	6.125642000	0.365324000	0.000000000
6	6.057085000	-0.984293000	0.000000000
6	5.744655000	-2.157650000	0.000000000
6	5.133009000	-3.362663000	0.000000000
6	4.370267000	-4.307446000	0.000000000
6	3.321361000	-5.159475000	0.000000000
6	-0.276942000	-6.129639000	0.000000000
6	0.935533000	-6.064151000	0.000000000
6	2.807749000	5.455943000	0.000000000
6	-2.746320000	-5.487163000	0.000000000

6	0.486541000	5.709995000	0.000000000
6	-5.458911000	1.744966000	0.000000000
6	-4.897773000	2.975848000	0.000000000
6	-3.991136000	-4.112578000	0.000000000
6	3.991136000	4.112578000	0.000000000
6	-2.039194000	5.355601000	0.000000000
6	2.039194000	-5.355601000	0.000000000

6	-1.612002000	-5.920420000	0.000000000
6	-6.067683000	0.916156000	0.000000000
6	-6.129331000	-0.296519000	0.000000000
6	-2.175992000	5.737249000	0.000000000
6	2.240294000	-5.712363000	0.000000000
6	-3.880885000	-4.753051000	0.000000000
6	3.827199000	4.796315000	0.000000000
6	-4.740943000	-3.895910000	0.000000000
6	4.784326000	3.842342000	0.000000000

Monoanions of cyclo[n]carbons

C16

Gas-phase. wB97XD/ma-def2-TZVPP

Atom	X	Y	Z
6	1.801633000	-2.827176000	0.000000000
6	0.597074000	-3.147563000	-0.000039000
6	-0.725168000	-3.273057000	-0.000078000
6	-1.803467000	-2.647858000	-0.000096000
6	-3.273058000	0.725169000	-0.000078000
6	-1.801633000	2.827176000	0.000000000
6	-0.597074000	3.147563000	0.000039000
6	0.725168000	3.273057000	0.000078000
6	1.803468000	2.647858000	0.000096000
6	2.647858000	-1.803467000	0.000042000
6	3.273058000	-0.725168000	0.000078000
6	-2.647858000	1.803467000	-0.000042000
6	3.147564000	0.597074000	0.000097000
6	-2.827176000	-1.801633000	-0.000110000
6	2.827177000	1.801633000	0.000109000
6	-3.147564000	-0.597074000	-0.000098000

C18

Gas-phase. wB97XD/ma-def2-TZVPP

Atom	X	Y	Z
6	0.671325000	3.529768000	0.000446000
6	-0.677118000	3.528851000	0.000404000
6	-1.823072000	3.099731000	0.000316000
6	-2.928528000	2.331299000	0.000188000
6	-3.496982000	-1.220366000	-0.000258000
6	-0.629399000	-3.489358000	-0.000441000
6	0.635194000	-3.488677000	-0.000401000
6	1.851857000	-3.034901000	-0.000307000
6	2.952828000	-2.418423000	-0.000198000
6	2.924516000	2.335640000	0.000374000
6	3.484326000	1.239850000	0.000260000
6	-2.948629000	-2.422655000	-0.000386000
6	3.895689000	-0.034559000	0.000119000
6	-3.486440000	1.234515000	0.000039000
6	3.499050000	-1.215101000	-0.000035000
6	-3.895799000	-0.040514000	-0.000128000
6	1.817969000	3.102515000	0.000431000
6	-1.846789000	-3.037615000	-0.000425000

C20

Gas-phase. wB97XD/ma-def2-TZVPP

Atom	X	Y	Z
6	1.164120000	-3.934789000	-0.014832000
6	2.369511000	-3.352802000	-0.010078000
6	3.254529000	-2.498974000	-0.008245000
6	3.887654000	-1.319651000	-0.001053000
6	4.101838000	-0.108697000	0.001364000
6	3.920909000	1.217539000	0.008297000
6	2.456518000	3.289746000	0.014431000
6	3.382440000	2.323137000	0.009788000
6	1.371014000	3.867661000	0.014906000
6	-1.164120000	3.934789000	0.014832000
6	-2.369511000	3.352802000	0.010078000
6	-3.254529000	2.498974000	0.008245000
6	-3.887654000	1.319651000	0.001053000
6	-4.101838000	0.108697000	-0.001364000
6	-3.920909000	-1.217539000	-0.008297000
6	-3.382440000	-2.323137000	-0.009788000
6	-2.456518000	-3.289746000	-0.014431000
6	-0.053745000	-4.105359000	-0.015136000

C22

Gas-phase. wB97XD/ma-def2-TZVPP

Atom	X	Y	Z
6	-3.456599000	-3.036592000	0.000020000
6	-1.192362000	-4.218085000	0.000017000
6	0.057060000	-4.332056000	0.000013000
6	1.345801000	-4.125553000	0.000008000
6	2.531858000	-3.721211000	0.000003000
6	3.482222000	-2.809646000	-0.000003000
6	4.529663000	-0.553262000	-0.000013000
6	4.293609000	-1.866102000	-0.000008000
6	4.614593000	0.675702000	-0.000017000
6	3.441124000	2.950198000	-0.000020000
6	1.238440000	4.242922000	-0.000017000
6	-0.104313000	4.397169000	-0.000013000
6	-1.307452000	4.202891000	-0.000009000
6	-2.551088000	3.675710000	-0.000003000
6	-3.449934000	2.849027000	0.000002000
6	-4.257388000	1.770997000	0.000008000
6	-4.519707000	0.571808000	0.000013000
6	-4.118107000	-1.888595000	0.000019000

6	-1.371014000	-3.867661000	-0.014906000
6	0.053745000	4.105359000	0.015136000

C24

Gas-phase. wB97XD/ma-def2-TZVPP

Atom	X	Y	Z
6	4.237674000	-2.476735000	0.000089000
6	3.422892000	-3.530774000	0.000055000
6	-0.027756000	-4.907926000	-0.000061000
6	-1.342908000	-4.719698000	-0.000098000
6	-2.480808000	-4.241441000	-0.000122000
6	-3.523988000	-3.415848000	-0.000140000
6	-4.761921000	-1.196520000	-0.000146000
6	-4.273720000	-2.437959000	-0.000148000
6	-4.923379000	0.021967000	-0.000134000
6	-4.252681000	2.477465000	-0.000086000
6	-3.416393000	3.527006000	-0.000050000
6	-2.442158000	4.267663000	-0.000015000
6	-1.192937000	4.764224000	0.000025000
6	0.020779000	4.912536000	0.000059000
6	1.352518000	4.725418000	0.000094000
6	2.475435000	4.240677000	0.000118000
6	3.538252000	3.418922000	0.000139000
6	4.776534000	1.194125000	0.000148000
6	4.265003000	2.433124000	0.000146000
6	-4.722895000	1.345135000	-0.000113000
6	4.733922000	-1.350693000	0.000112000
6	4.910143000	-0.025980000	0.000135000
6	1.198347000	-4.762290000	-0.000024000
6	2.430047000	-4.262400000	0.000017000

C28

Gas-phase. wB97XD/ma-def2-TZVPP

Atom	X	Y	Z
6	2.474385000	-5.101842000	0.000000000
6	3.638219000	-4.416175000	0.000000000
6	5.625256000	-1.271199000	0.000000000
6	5.848326000	0.052616000	0.000000000
6	5.633402000	1.261528000	0.000000000
6	5.250918000	2.540213000	0.000000000
6	2.480858000	5.049169000	0.000000000
6	3.582184000	4.476419000	0.000000000
6	1.238338000	5.485774000	0.000000000
6	-2.502149000	5.113055000	0.000000000
6	-3.608557000	4.389902000	0.000000000
6	-4.559433000	3.598053000	0.000000000
6	-5.217539000	2.440489000	0.000000000
6	-5.704406000	1.310804000	0.000000000
6	-5.775653000	-0.027526000	0.000000000
6	-5.682247000	-1.247781000	0.000000000

6	-4.634045000	-0.762516000	0.000017000
6	4.090951000	1.913574000	-0.000019000
6	-2.387406000	-3.684789000	0.000019000
6	2.353078000	3.748409000	-0.000019000

C26

Gas-phase. wB97XD/ma-def2-TZVPP

Atom	X	Y	Z
6	4.354777000	3.058648000	-0.000423000
6	5.074148000	2.051805000	-0.000449000
6	5.050650000	-1.768322000	-0.000092000
6	4.533648000	-2.875361000	0.000005000
6	3.605706000	-3.854437000	0.000189000
6	2.623178000	-4.574681000	0.000297000
6	-1.193445000	-5.099578000	0.000520000
6	0.153121000	-5.223015000	0.000476000
6	-2.339950000	-4.690892000	0.000500000
6	-4.315679000	-3.090154000	0.000395000
6	-5.044134000	-1.956952000	0.000283000
6	-5.318881000	-0.764095000	0.000206000
6	-5.450196000	0.570225000	0.000072000
6	-5.079993000	1.745068000	0.000024000
6	-4.558433000	2.969219000	-0.000052000
6	-3.644576000	3.809424000	-0.000128000
6	-2.591103000	4.600795000	-0.000169000
6	1.142997000	5.063971000	-0.000386000
6	-0.102977000	5.157493000	-0.000366000
6	-3.497063000	-3.993969000	0.000457000
6	3.489552000	4.059443000	-0.000496000
6	2.378503000	4.623123000	-0.000412000
6	5.439011000	-0.481855000	-0.000288000
6	5.333014000	0.741770000	-0.000317000
6	1.357354000	-5.048587000	0.000415000
6	-1.399229000	4.970913000	-0.000260000

C30

Gas-phase. wB97XD/ma-def2-TZVPP

Atom	X	Y	Z
6	3.645638000	4.873386000	0.000000000
6	2.601171000	5.497234000	0.000001000
6	-1.240655000	5.931522000	0.000001000
6	-2.401067000	5.568208000	0.000001000
6	-3.595392000	4.936248000	0.000000000
6	-4.505006000	4.127178000	0.000000000
6	-6.212910000	0.670156000	-0.000001000
6	-5.845863000	1.963425000	-0.000001000
6	-6.173686000	-0.554274000	-0.000001000
6	-3.687197000	-4.822984000	0.000001000
6	-2.559976000	-5.505635000	0.000001000
6	-1.358096000	-5.831462000	0.000001000
6	-0.054978000	-5.983456000	0.000001000
6	1.188089000	-5.890664000	0.000001000
6	2.441793000	-5.498258000	0.000001000
6	3.573870000	-4.984209000	0.000000000

6	-5.167275000	-2.491652000	0.000000000
6	-2.509497000	-5.097081000	0.000000000
6	-3.538975000	-4.447598000	0.000000000
6	0.000000000	5.605548000	0.000000000
6	-0.016466000	-5.653532000	0.000000000
6	-1.225454000	-5.519901000	0.000000000
6	5.258310000	-2.437028000	0.000000000
6	4.488707000	-3.543387000	0.000000000
6	4.498968000	3.520030000	0.000000000
6	-4.531320000	-3.532319000	0.000000000
6	1.327980000	-5.509694000	0.000000000
6	-1.306880000	5.453115000	0.000000000

6	4.548803000	-4.091165000	0.000000000
6	6.224058000	-0.760422000	-0.000002000
6	5.878357000	-1.939525000	-0.000001000
6	-5.983361000	-1.878244000	0.000000000
6	5.959709000	1.784701000	-0.000001000
6	6.163146000	0.579073000	-0.000002000
6	0.106242000	6.050894000	0.000001000
6	1.313686000	5.909272000	0.000001000
6	-5.356540000	3.081257000	-0.000001000
6	5.379406000	-3.175166000	-0.000001000
6	5.363933000	2.992265000	0.000000000
6	-5.405852000	-2.966559000	0.000000000
6	4.670333000	3.994427000	0.000000000
6	-4.677655000	-4.077223000	0.000000000

Dianions of cyclo[n]carbons

C16 singlet
Gas-phase. wB97XD/ma-def2-TZVPP

Atom	X	Y	Z
6	1.499006000	2.803147000	0.000079000
6	2.604601000	2.136737000	-0.000047000
6	3.042076000	0.922174000	-0.000111000
6	3.352603000	-0.330824000	-0.000110000
6	0.922174000	-3.042073000	-0.000117000
6	-1.499006000	-2.803147000	0.000037000
6	-2.604601000	-2.136737000	0.000048000
6	-3.042076000	-0.922174000	0.000059000
6	-3.352603000	0.330824000	0.000115000
6	0.330824000	3.352598000	0.000036000
6	-0.922174000	3.042073000	0.000009000
6	-0.330824000	-3.352598000	-0.000030000
6	-2.136737000	2.604599000	0.000099000
6	2.803150000	-1.499005000	-0.000078000
6	-2.803150000	1.499005000	0.000116000
6	2.136737000	-2.604599000	-0.000106000

C16 triplet
Gas-phase. wB97XD/ma-def2-TZVPP

Atom	X	Y	Z
6	0.907593000	3.059358000	-0.000005000
6	2.038409000	2.569042000	-0.000045000
6	2.964231000	1.584069000	-0.000083000
6	3.307889000	0.389263000	-0.000102000
6	1.599693000	-2.805800000	-0.000075000
6	-0.891415000	-3.014475000	0.000004000
6	-2.126002000	-2.627433000	0.000046000
6	-2.828479000	-1.531365000	0.000078000
6	-3.454716000	-0.414843000	0.000107000
6	-0.432097000	3.261169000	0.000040000
6	-1.564763000	2.756356000	0.000073000
6	0.377546000	-3.219982000	-0.000039000
6	-2.716077000	2.060558000	0.000104000
6	3.327098000	-0.941874000	-0.000112000
6	-3.112526000	0.866153000	0.000107000
6	2.603617000	-1.990196000	-0.000099000

C18 singlet
Gas-phase. wB97XD/ma-def2-TZVPP

Atom	X	Y	Z
6	-1.843358000	2.854638000	0.000429000
6	-3.043802000	2.266086000	0.000460000
6	-3.646551000	1.164456000	0.000344000
6	-4.267653000	-0.009884000	0.000232000
6	-1.852353000	-2.838338000	-0.000232000
6	1.849687000	-2.839246000	-0.000425000
6	3.045403000	-2.285959000	-0.000424000
6	3.650216000	-1.163284000	-0.000326000
6	4.268077000	-0.012855000	-0.000219000
6	0.670861000	3.239099000	0.000315000
6	1.845847000	2.853264000	0.000215000
6	-0.661009000	-3.223706000	-0.000335000
6	3.045908000	2.264030000	0.000094000
6	-3.651223000	-1.160984000	0.000060000
6	3.647665000	1.161821000	-0.000061000

C18 triplet
Gas-phase. wB97XD/ma-def2-TZVPP

Atom	X	Y	Z
6	0.761019000	3.442828000	0.000380000
6	-0.568038000	3.557095000	0.000438000
6	-1.725046000	3.090460000	0.000349000
6	-2.894354000	2.464570000	0.000245000
6	-3.515824000	-1.112819000	-0.000293000
6	-0.796427000	-3.523620000	-0.000434000
6	0.538530000	-3.474052000	-0.000368000
6	1.738154000	-3.152382000	-0.000302000
6	2.843411000	-2.414982000	-0.000203000
6	3.002142000	2.253271000	0.000324000
6	3.585903000	1.133413000	0.000272000
6	-3.056728000	-2.298094000	-0.000417000
6	3.944964000	-0.115651000	0.000152000
6	-3.434691000	1.315807000	0.000054000
6	3.503371000	-1.342801000	-0.000025000

6	-3.047829000	-2.284495000	-0.000099000
6	-0.668021000	3.239303000	0.000378000
6	0.658136000	-3.223946000	-0.000407000

6	-3.944458000	0.116404000	-0.000136000
6	1.940057000	3.046349000	0.000426000
6	-1.921986000	-2.985798000	-0.000459000

C20 singlet

Gas-phase. wB97XD/ma-def2-TZVPP

Atom	X	Y	Z
6	3.275144000	-2.351297000	0.000000000
6	2.439748000	-3.358047000	0.000000000
6	1.267624000	-3.827376000	0.000000000
6	0.000013000	-4.150792000	0.000000000
6	-1.224113000	-3.841513000	0.000000000
6	-2.439725000	-3.358065000	0.000000000
6	-3.947525000	-1.282667000	0.000000000
6	-3.248255000	-2.388310000	0.000000000
6	-4.031649000	-0.022876000	0.000000000
6	-3.275144000	2.351297000	0.000000000
6	-2.439748000	3.358047000	0.000000000
6	-1.267624000	3.827376000	0.000000000
6	-0.000013000	4.150792000	0.000000000
6	1.224113000	3.841513000	0.000000000
6	2.439725000	3.358065000	0.000000000
6	3.248255000	2.388310000	0.000000000
6	3.947525000	1.282667000	0.000000000
6	3.947525000	-1.282628000	0.000000000
6	4.031649000	0.022876000	0.000000000
6	-3.947525000	1.282628000	0.000000000

C20 triplet

Gas-phase. wB97XD/ma-def2-TZVPP

Atom	X	Y	Z
6	3.767820000	1.965028000	0.000000000
6	4.115963000	0.693586000	0.000000000
6	4.260718000	-0.547333000	0.000000000
6	3.735313000	-1.779053000	0.000000000
6	3.042037000	-2.799141000	0.000000000
6	1.911385000	-3.537336000	0.000000000
6	-0.609328000	-3.962641000	0.000000000
6	0.742032000	-3.907654000	0.000000000
6	-1.771951000	-3.563526000	0.000000000
6	-3.634961000	-1.882310000	0.000000000
6	-4.251334000	-0.700390000	0.000000000
6	-4.104230000	0.549214000	0.000000000
6	-3.859082000	1.838986000	0.000000000
6	-2.960901000	2.743158000	0.000000000
6	-1.961827000	3.565027000	0.000000000
6	-0.716745000	3.864689000	0.000000000
6	0.569386000	3.916960000	0.000000000
6	2.872931000	2.862245000	0.000000000
6	1.807049000	3.599856000	0.000000000
6	-2.954277000	-2.919368000	0.000000000

C22 singlet

Gas-phase. wB97XD/ma-def2-TZVPP

Atom	X	Y	Z
6	-1.549625000	-3.760869000	-0.000019000
6	-3.739655000	-2.528690000	-0.000017000
6	-4.687410000	-1.655194000	-0.000015000
6	-4.783728000	-0.343642000	-0.000010000
6	-4.910077000	0.911246000	-0.000005000
6	-4.137219000	2.006292000	0.000001000
6	-2.180732000	3.590613000	0.000011000
6	-3.385285000	2.986413000	0.000006000
6	-1.021547000	3.986591000	0.000015000
6	1.529722000	3.829773000	0.000020000
6	3.702751000	2.533257000	0.000018000
6	4.628621000	1.560747000	0.000016000
6	4.780008000	0.317692000	0.000010000
6	4.962870000	-0.995165000	0.000005000
6	4.173392000	-2.008519000	-0.000001000
6	3.392327000	-3.030603000	-0.000008000
6	2.200163000	-3.534309000	-0.000012000
6	-0.302572000	-3.982240000	-0.000018000
6	0.993076000	-3.923500000	-0.000016000
6	0.327399000	4.058335000	0.000018000
6	-2.789300000	-3.385359000	-0.000020000
6	2.796821000	3.367132000	0.000020000

C22 triplet

Gas-phase. wB97XD/ma-def2-TZVPP

Atom	X	Y	Z
6	-1.298786000	4.288782000	0.000000000
6	1.255636000	4.146848000	0.000000000
6	2.429660000	3.591177000	0.000000000
6	3.406618000	2.779092000	0.000000000
6	4.201025000	1.763946000	0.000000000
6	4.539510000	0.520494000	0.000000000
6	4.191137000	-1.959208000	0.000000000
6	4.690823000	-0.740830000	0.000000000
6	3.560732000	-3.034472000	0.000000000
6	1.312784000	-4.253701000	0.000000000
6	-1.236625000	-4.132020000	0.000000000
6	-2.485642000	-3.616426000	0.000000000
6	-3.381469000	-2.774173000	0.000000000
6	-4.241495000	-1.744668000	0.000000000
6	-4.500248000	-0.526605000	0.000000000
6	-4.695766000	0.781621000	0.000000000
6	-4.157281000	1.929891000	0.000000000
6	-2.444845000	3.729039000	0.000000000
6	-3.558603000	3.080806000	0.000000000
6	2.446750000	-3.778384000	0.000000000
6	0.000000000	4.309224000	0.000000000
6	-0.033916000	-4.360432000	0.000000000

C24 singlet

Gas-phase. wB97XD/ma-def2-TZVPP

Atom	X	Y	Z
6	-4.148739000	-2.698511000	0.000175000
6	-3.252930000	-3.633526000	0.000390000
6	0.459240000	-4.461322000	0.000773000
6	1.714378000	-4.207938000	0.000814000
6	2.931942000	-3.811700000	0.000829000
6	3.901942000	-2.958218000	0.000751000
6	5.095104000	-0.801772000	0.000470000
6	4.837560000	-2.094627000	0.000668000
6	5.352774000	0.425556000	0.000282000
6	4.241125000	2.746909000	-0.000178000
6	3.164052000	3.561357000	-0.000384000
6	2.111651000	4.184098000	-0.000557000
6	0.791183000	4.489581000	-0.000695000
6	-0.427522000	4.531742000	-0.000783000
6	-1.763586000	4.301859000	-0.000832000
6	-2.858195000	3.758665000	-0.000814000
6	-3.984962000	3.012750000	-0.000764000
6	-5.283339000	0.791034000	-0.000480000
6	-4.631999000	1.964884000	-0.000633000
6	4.794831000	1.644518000	0.000042000
6	-5.015209000	-1.768040000	-0.000037000
6	-5.147875000	-0.452335000	-0.000265000
6	-0.822761000	-4.431430000	0.000684000
6	-2.058667000	-4.093534000	0.000546000

C24 triplet

Gas-phase. wB97XD/ma-def2-TZVPP

Atom	X	Y	Z
6	1.149929000	4.660112000	-0.000001000
6	-0.196841000	4.773570000	0.000204000
6	-3.603534000	3.296134000	0.000659000
6	-4.488293000	2.289383000	0.000754000
6	-4.863353000	1.107362000	0.000767000
6	-5.118655000	-0.193254000	0.000758000
6	-4.297589000	-2.616939000	0.000547000
6	-4.779038000	-1.401542000	0.000663000
6	-3.379203000	-3.491392000	0.000377000
6	-1.112713000	-4.621151000	-0.000003000
6	0.167189000	-4.745169000	-0.000199000
6	1.432288000	-4.574669000	-0.000382000
6	2.605788000	-4.041635000	-0.000538000
6	3.669051000	-3.344305000	-0.000670000
6	4.394445000	-2.264343000	-0.000739000
6	4.978480000	-1.147321000	-0.000785000
6	4.998762000	0.171317000	-0.000740000
6	4.210444000	2.563121000	-0.000535000
6	4.886982000	1.411808000	-0.000678000
6	-2.332933000	-4.250073000	0.000193000
6	2.296177000	4.228971000	-0.000188000
6	3.448001000	3.531232000	-0.000386000
6	-2.661534000	4.085049000	0.000547000
6	-1.403850000	4.573734000	0.000377000

C26 singlet

Gas-phase. wB97XD/ma-def2-TZVPP

Atom	X	Y	Z
6	0.603542000	4.824820000	0.000000000
6	-0.616619000	4.823472000	0.000000000
6	-4.192647000	3.339883000	0.000000000
6	-4.915636000	2.343422000	0.000000000
6	-5.652605000	1.225501000	0.000000000
6	-5.672028000	-0.023146000	0.000000000
6	-4.210325000	-3.422426000	0.000000000
6	-4.962485000	-2.370516000	0.000000000
6	-3.069738000	-4.010243000	0.000000000
6	-0.629215000	-4.715409000	0.000000000
6	0.642222000	-4.714115000	0.000000000
6	1.915842000	-4.524313000	0.000000000
6	3.081275000	-4.004252000	0.000000000
6	4.220701000	-3.414259000	0.000000000
6	4.969623000	-2.359887000	0.000000000
6	5.719048000	-1.331902000	0.000000000
6	5.671852000	-0.010417000	0.000000000
6	4.182699000	3.348583000	0.000000000
6	4.908441000	2.354204000	0.000000000
6	-1.903135000	-4.527552000	0.000000000
6	1.933381000	4.582731000	0.000000000
6	3.038617000	4.059967000	0.000000000
6	-3.050024000	4.053546000	0.000000000

C26 triplet

Gas-phase. wB97XD/ma-def2-TZVPP

Atom	X	Y	Z
6	5.421197000	0.481252000	0.000000000
6	5.243647000	1.715572000	0.000000000
6	2.604309000	4.500846000	0.000000000
6	1.472394000	4.963298000	0.000000000
6	0.130299000	5.136434000	0.000000000
6	-1.085518000	5.028753000	0.000000000
6	-4.429867000	3.110982000	0.000000000
6	-3.414916000	3.990018000	0.000000000
6	-5.005303000	2.016180000	0.000000000
6	-5.421851000	-0.467039000	0.000000000
6	-5.230405000	-1.759703000	0.000000000
6	-4.534773000	-2.822274000	0.000000000
6	-3.721349000	-3.821239000	0.000000000
6	-2.619425000	-4.475282000	0.000000000
6	-1.436438000	-4.956467000	0.000000000
6	-0.159836000	-5.116451000	-0.000001000
6	1.109691000	-5.004513000	0.000000000
6	4.403908000	-3.143928000	0.000000000
6	3.425558000	-3.966056000	0.000000000
6	-5.498241000	0.784170000	0.000000000
6	5.492424000	-0.831268000	0.000000000
6	5.008428000	-1.999332000	0.000000000
6	3.753409000	3.798257000	0.000000000

6	-1.945994000	4.578914000	0.000000000
6	-5.714952000	-1.344643000	0.000000000
6	5.648160000	1.238037000	0.000000000
6	4.529447000	2.841663000	0.000000000
6	-2.383598000	4.651603000	0.000000000
6	2.346810000	-4.655477000	0.000000000

C28 singlet

Gas-phase. wB97XD/ma-def2-TZVPP

Atom	X	Y	Z
6	3.510398000	4.305126000	0.000312000
6	4.602150000	3.515690000	0.000245000
6	6.022619000	0.098413000	-0.000025000
6	6.075122000	-1.225498000	-0.000127000
6	5.444561000	-2.316649000	-0.000207000
6	4.791415000	-3.444630000	-0.000290000
6	1.381448000	-5.103473000	-0.000398000
6	2.622962000	-4.801837000	-0.000382000
6	0.109522000	-5.250166000	-0.000403000
6	-3.587387000	-4.352960000	-0.000315000
6	-4.547726000	-3.487871000	-0.000243000
6	-5.468499000	-2.615907000	-0.000173000
6	-5.843015000	-1.357879000	-0.000074000
6	-6.205514000	-0.158338000	0.000020000
6	-5.869199000	1.130296000	0.000118000
6	-5.527067000	2.318364000	0.000207000
6	-4.651524000	3.336557000	0.000281000
6	-1.423101000	5.207815000	0.000408000
6	-2.576171000	4.808331000	0.000383000
6	-1.167045000	-5.172018000	-0.000390000
6	1.136952000	5.244055000	0.000397000
6	-0.077686000	5.349237000	0.000412000
6	5.964458000	1.339765000	0.000070000
6	5.285204000	2.493665000	0.000163000
6	3.730513000	-4.146359000	-0.000338000
6	-3.780162000	4.198420000	0.000342000
6	2.445659000	4.904685000	0.000364000
6	-2.398885000	-4.816835000	-0.000356000

C28 triplet

Gas-phase. wB97XD/ma-def2-TZVPP

Atom	X	Y	Z
6	2.590828000	-5.015265000	0.000000000
6	3.669530000	-4.310489000	0.000000000
6	5.783186000	-1.202119000	0.000000000
6	5.825707000	0.112328000	0.000000000
6	5.741713000	1.354625000	0.000000000
6	5.174950000	2.558554000	0.000000000
6	2.440198000	5.094062000	0.000000000
6	3.488696000	4.461697000	0.000000000
6	1.143599000	5.469773000	0.000000000
6	-2.561287000	5.005741000	0.000000000
6	-3.731197000	4.339234000	0.000000000
6	-4.575924000	3.446846000	0.000000000
6	-5.360968000	2.365517000	0.000000000
6	-5.696454000	1.172079000	0.000000000
6	-5.918800000	-0.130913000	0.000000000
6	-5.649998000	-1.354723000	0.000000000
6	-5.244479000	-2.596002000	0.000000000
6	-2.391905000	-5.062787000	0.000000000
6	-3.525747000	-4.483782000	0.000000000
6	-0.071990000	5.583249000	0.000000000
6	0.101981000	-5.563773000	0.000000000
6	-1.170452000	-5.461189000	0.000000000
6	5.275896000	-2.353674000	0.000000000
6	4.630540000	-3.481864000	0.000000000
6	4.522318000	3.604493000	0.000000000
6	-4.450483000	-3.580777000	0.000000000
6	1.374798000	-5.399007000	0.000000000
6	-1.414255000	5.428165000	0.000000000

C30 singlet

Gas-phase. wB97XD/ma-def2-TZVPP

Atom	X	Y	Z
6	4.305330000	4.190974000	-0.001237000
6	3.306611000	4.896313000	-0.001044000
6	-0.500529000	5.662164000	-0.000409000
6	-1.702784000	5.462106000	-0.000196000
6	-2.988731000	5.049010000	0.000080000
6	-4.024941000	4.401366000	0.000286000
6	-6.432704000	1.392527000	0.001006000
6	-5.759155000	2.547602000	0.000757000
6	-6.510612000	0.152396000	0.000804000
6	-4.358586000	-4.173289000	0.000775000
6	-3.293063000	-4.889598000	0.000755000
6	-2.066962000	-5.258989000	0.000783000
6	-0.816293000	-5.511000000	0.000713000
6	0.466632000	-5.534293000	0.000725000
6	1.727541000	-5.343088000	0.000581000

C30 triplet

Gas-phase. wB97XD/ma-def2-TZVPP

Atom	X	Y	Z
6	-4.200889000	4.411982000	0.000001000
6	-3.220314000	5.145788000	0.000001000
6	0.573336000	5.940530000	-0.000001000
6	1.771043000	5.711657000	-0.000002000
6	3.037340000	5.243154000	-0.000002000
6	4.042205000	4.543992000	-0.000003000
6	6.184949000	1.355709000	-0.000003000
6	5.647073000	2.569673000	-0.000003000
6	6.258046000	0.110439000	-0.000003000
6	4.207427000	-4.388036000	-0.000001000
6	3.178808000	-5.154974000	-0.000001000
6	1.989845000	-5.629409000	0.000000000
6	0.743885000	-5.909932000	0.000001000
6	-0.538576000	-5.928591000	0.000001000
6	-1.792902000	-5.689393000	0.000002000

6	2.967758000	-5.021880000	0.000552000	6	-2.995818000	-5.250507000	0.000002000
6	4.072445000	-4.370797000	0.000300000	6	-4.049663000	-4.519787000	0.000003000
6	6.508805000	-1.512981000	-0.000752000	6	-6.170309000	-1.401413000	0.000003000
6	5.830793000	-2.578775000	-0.000320000	6	-5.646614000	-2.550333000	0.000003000
6	-6.596527000	-1.168396000	0.000568000	6	6.218508000	-1.199894000	-0.000003000
6	6.534125000	1.052744000	-0.001363000	6	-6.231133000	1.153494000	0.000003000
6	6.517128000	-0.192058000	-0.001032000	6	-6.258004000	-0.094763000	0.000003000
6	0.851391000	5.631490000	-0.000654000	6	-0.779511000	5.920561000	-0.000001000
6	2.041663000	5.368342000	-0.000840000	6	-1.969180000	5.652149000	0.000000000
6	-5.089404000	3.578401000	0.000557000	6	5.033607000	3.640235000	-0.000003000
6	5.145203000	-3.681752000	0.000026000	6	-4.998392000	-3.669870000	0.000003000
6	5.928849000	2.243382000	-0.001412000	6	-5.736401000	2.384338000	0.000002000
6	-5.997058000	-2.277635000	0.000700000	6	5.735665000	-2.365465000	-0.000002000
6	5.326880000	3.316807000	-0.001483000	6	-5.162115000	3.477482000	0.000002000
6	-5.393804000	-3.431091000	0.000774000	6	5.128084000	-3.508816000	-0.000002000

References

[1] J.-D. Chai, M. Head-Gordon, Long-range corrected hybrid density functionals with damped atom–atom dispersion corrections, *Phys. Chem. Chem. Phys.* 2008, 10, 6615-6620.

[2] F. Weigend, R. Ahlrichs, Balanced basis sets of split valence, triple zeta valence and quadruple zeta valence quality for H to Rn: Design and assessment of accuracy, *Phys. Chem. Chem. Phys.* 2005, 7, 3297-3305.

[3] J. Zheng, X. Xu, D.G. Truhlar, Minimally augmented Karlsruhe basis sets, *Theor. Chem. Acc.* 2011, 128, 295-305.

[4] G. A. Zhurko, Chemcraft 1.80 (build 523b) - graphical program for visualization of quantum chemistry computations. https://chemcraftprog.com/

[5] D.W. Szczepanik, A new perspective on quantifying electron localization and delocalization in molecular systems, *Comput. Theor. Chem.* 2016, 1080, 33–37.

[6] D.W. Szczepanik, M. Andrzejak, K. Dyduch, E. Żak, M. Makowski, G. Mazur and J. Mrozek, A uniform approach to the description of multicenter bonding, *Phys. Chem. Chem. Phys.* 2014, 16, 20514-20523.

[7] D. W. Szczepanik, and M. Solà, The Electron Density of Delocalized Bonds (EDDBs) as a Measure of Local and Global Aromaticity. In Aromaticity; Elsevier, 2021; pp 259–284.

[8] E. Matito, An electronic aromaticity index for large rings, *Phys. Chem. Chem. Phys.* 2016, 18, 11839–11846.
